# Optimization of Water Lentil (Duckweed) Leaf Protein Purification: Identification, Structure, and Foaming Properties

**DOI:** 10.3390/foods12183424

**Published:** 2023-09-14

**Authors:** Tristan Muller, Marie-Ève Bernier, Laurent Bazinet

**Affiliations:** 1 Institute of Nutrition and Functional Foods (INAF), Department of Food Sciences, Université Laval, Quebec, QC G1V 0A6, Canada; tristan.muller.1@ulaval.ca (T.M.); marie-eve.bernier.7@ulaval.ca (M.-È.B.); 2 Laboratoire de Transformation Alimentaire et Procédés Électro Membranaires (LTAPEM), Laboratory of Food Processing and Electro Membrane Processes, Université Laval, Quebec, QC G1V 0A6, Canada

**Keywords:** water lentil proteins, extraction, purification, identification, structure, solubility, foaming properties

## Abstract

Water lentil, commonly known as duckweed, is an aquatic plant with great agronomic potential, as it can double its biomass in less than 24 h and contains up to 45% leaf proteins on a dry matter basis. However, extracting proteins from leaves is an arduous process due to the complexity of the matrix, which limits their uses in the food industry. In this study, water lentil protein extraction by solubilization was maximized using response surface methodology. By heating at 80 °C at pH 11 with a water lentil powder concentration of 2% or 4% for 2 h, up to 77.8% of total proteins were solubilized. Then, by precipitating the solubilized proteins at pH 4, a protein purity of 57.6% combined with a total protein yield of 60.0% was achieved. To the best of our knowledge, this is the highest leaf protein extraction yield reported in the literature with such protein purity. Proteomics analyses showed that the protein concentrate was composed of around 85.0% RubisCO, and protein structure analyses using ATR-FTIR and DSC were linked to a high protein solubility in water at pH 7. Moreover, a 1.5% protein solution of the protein concentrate at pH 7 showed excellent foaming properties compared to a 10.3% protein egg white solution. It had a superior foaming capacity (194% vs. 122%, respectively) for the same foaming stability after 60 min, which confirms water lentil proteins’ potential for human nutrition and food formulation.

## 1. Introduction

Climate change has already been reported to be the main cause of substantial biodiversity, ecosystems, infrastructure, and human losses in the last few years [1], with temperatures having increased by 1.1 °C compared to pre-industrial levels. Faced with this issue, the food industry plays a major role since it contributes to nearly 16 billion tons of CO_2_ equivalent emissions per year, or 34% of global emissions [2]. Thus, the food sector has a key role in limiting CO_2_ emissions as it can promote low-impact, nutritious, and mass-scalable ingredients. The consumption of plant-based foods to replace animal-based products in the human diet is one of the solutions to achieve these goals [3], especially regarding protein intake.

In the last decades, proteins from seeds or plants such as soy or pea have been thoroughly studied, showing promising applications in functional and new food products [4,5], considering their low environmental impact compared to animal-based proteins [6]. However, plant-based proteins are generally considered lower-quality proteins compared to animal-based proteins due to their incomplete amino-acid profile (with relatively low lysine content for cereals [7], or their lower digestibility [8]. Thus, it is generally accepted that a wide range of plant-based proteins should be consumed to meet human protein and amino acid requirements [9]. A new source of plant protein, water lentil, commonly known as duckweed, has been recently rediscovered after its popularization by the work of several authors [10,11,12] in the 1980s. Water lentils are free-floating aquatic plants that can grow almost anywhere on Earth. As with many other leaves, water lentils are very rich in RubisCO [13], a protein that showed promising properties for human consumption, such as being tasteless, odorless, white-colored, having a high nutritive value, and having interesting functional properties [14]. Water lentils have great agronomic potential since they can double their biomass in less than 24 h. Hence, 13–38 t of dry matter (DM) per ha and per year [15] and 200–350 g of protein/kg of DM can be produced [16] In another study, a yield of 10–18 t protein/ha/year was found, compared to 0.6–1.2 t protein/ha/year for soy in Europe [17]. The potential uses of water lentils have already been extensively explored for energy production, water purification, and animal feed [15]. However, their use in human food is still limited, mainly due to the protein extraction step.

The extraction of proteins from leaves such as alfalfa, spinach, or sugar beet leaves [18] usually consists of three steps. (1) First, the leaves’ cell walls are disrupted using mechanical treatments to release the soluble proteins from the cell’s compartments. A green juice containing the proteins of interest and a fibrous pulp are thus produced [19]. The green juice usually consists of around 50% to 70% of total proteins [20,21]. Some authors showed that both soluble and insoluble proteins are distributed evenly over these two products [22], meaning that this separation method may lead to inefficient protein separation. (2) Then, the green juice is commonly heated at around 50 °C to 55 °C [18]. Additionally, the pH of green juice can be increased up to pH 8–11 to improve protein solubilization [23,24,25,26,27]. After centrifugation, the proteins contained in the green juice are fractionated by separation of the soluble proteins (white fractions in the supernatant) from the chlorophyll and insoluble proteins (green fraction in the pellet). The white fraction usually contains around 40% of the total leaf protein. However, the obtained product has a low protein content as other soluble compounds remain in solution [20,21,22]. Tamayo Tenorio et al. [22] showed that the soluble and insoluble protein distributions of the white supernatant fraction and the green pellet fraction are almost even. Indeed, after washing the insoluble green pellet, they managed to obtain a product containing 41.1% proteins, which represented 26.7% of total leaf proteins, and a large quantity of RubisCO protein. Therefore, the production of the white fraction may also lead to unwanted RubisCO losses. (3) Finally, the proteins contained in the white fraction are purified by isolation of the soluble proteins from other water-soluble elements such as salts, sugars, or soluble fibers. The main methods for protein purification are protein coagulation by increasing temperature between 55 °C and 100 °C [26,27,28,29,30,31,32,33,34,35] or isoelectric precipitation by lowering the pH of the solution between 3 and 6 [24,25,26,27,28,29,30,31,32,33,34,35,36,37,38]. Other authors also used filtration steps in combination with temperature and/or pH changes, such as ultrafiltration [33,39,40,41,42] or diafiltration [26]. The final centrifugation step produces a white leaf protein concentrate (pellet commonly called LPC) and a brown juice (supernatant). The LPC usually has a protein purity of around 65% to 95% depending on the method used, since ion exchange chromatography produces very pure products (~95% protein), whereas heat increases, pH lowering, or filtration generally do not produce products with more than 70% protein purity [22]. However, such purification steps decrease the final protein yield [43], which is usually between 5% and 25% of total protein [20,42]. For water lentil, Rusoff et al. [11] were the first to extract its proteins by solubilization at pH 8.5 followed by their precipitation at pH 3.65, which led to a final product of 44.7% protein purity. Yu et al. [44] replicated their method and obtained a protein yield of around 50% and a purity of 67.8%. However, the protein concentrate obtained had poor solubility as well as lesser emulsifying properties. Nieuwland et al. [13] performed three different methods for protein extraction: heat treatment (70 °C for 60 min), isoelectric precipitation (pH 4.2), and precipitation using sodium metabisulfite, followed by mild heat treatment (52 °C for 33 min), followed by three filtrations. They eliminated the two first methods because they led to low purity and yield (heat treatment) or major difficulties to solubilize the concentrate (isoelectric point precipitation). The third method allowed them to obtain a concentrate with 67.2% protein purity and a total protein yield of 14.2%, which was mostly composed of the RubisCO protein. The LPC showed excellent solubility and gelling properties. Very recently, Duangjarus et al. [45] produced a LPC from duckweed using ultrasound-assisted extraction before isoelectric precipitation (pH 3.5), which showed interesting solubility and emulsifying properties; however, the protein yields and protein purity were not reported.

Therefore, the current production methods of LPCs are hampered by low protein yields, which need to be increased if LPCs are to be mass produced in the future [43]. Despite their great agronomic potential, this is even more true for water lentils, as there are still very few studies focusing on the extraction of their protein. Hence, in this context, the aim of the present study is to provide a simple and optimized method to extract water lentil proteins with high yield, purity, and functionality. To achieve this, the specific objectives are: (1) to maximize water lentil protein extraction and purification; (2) to identify the protein composition of the products at the initial and final steps of the process; (3) to study the structural changes of the proteins; and (4) to assess the foaming properties of the products at the initial and final steps of the process.

## 2. Materials and Methods

### 2.1. Materials

Water lentil powder was produced by the Lemnature (Vero Beach, Florida, USA) company and then provided by the Seta Organic company (Piedmont, QC, Canada). The water lentil powder was produced from freshly harvested water lentil leaves that were defatted, dried, and ground. The proximal composition of the water lentil powder is presented in Table 1 and is consistent with other water lentil proximal compositions reported in the literature. The total protein content, ash, dietary fibers, and moisture content were analyzed.

### 2.2. Methods

#### 2.2.1. Water Lentil Protein Extraction and Purification

The following sections describe three protocols. The first and second ones led to the optimization of water lentil protein extraction by solubilization. The third protocol led to the optimization of the purification by isoelectric point precipitation after the optimization of their solubilization.

##### Water Lentil Protein Extraction by Solubilization—First Protocol

A central composite design (CCD) was used to optimize water lentil protein extraction by solubilization. A CCD is a response surface methodology design, which is a combination of mathematical and statistical techniques used to optimize processes by generating a predictive quadratic model for the response variable [46]. Following the results of a preliminary central composite design (see Supplementary Material, Table S1, Figures S1 and S2), the solubilization of water lentil protein was optimized by the CCD presented in Table 2.

This CCD allowed us to test the effects of 3 concentrations (2-4-6% water lentil powder, *w*/*w*), 3 pH (9-10-11), and 3 temperatures (50-65-80 °C) on the quantity of protein solubilized as well as the purity of the obtained product. Such concentrations were chosen to replicate the composition of freshly harvested water lentils containing between 4% and 8% dry matter [16] (8% was replaced by 2% because of preliminary tests, Figure S1). Such temperatures (50, 65, and 80 °C) and pH values (9, 10, and 11) were chosen according to previous studies, and it was assumed that above 80 °C, energy consumption would be too high for a potential industrial scale-up. Therefore, 80 °C was chosen as the upper temperature limit. Moreover, it was difficult to increase the pH above pH 11 for highly concentrated solutions, even when using 5 M NaOH. Therefore, pH 11 was chosen as the upper pH limit.

**Figure 1:**
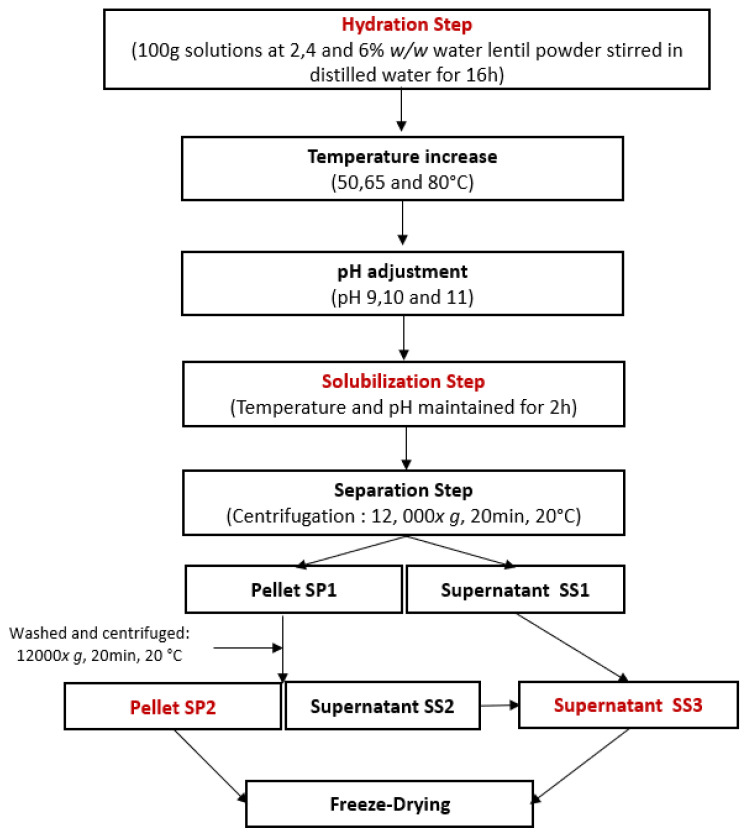
Experimental workflow for protein extraction by solubilization using a central composite design.

Following the workflow presented in Figure 1, water lentil powder was hydrated at a concentration of 2, 4, or 6% *w*/*w* in distilled water, and the solution was then gently stirred overnight at 4 °C to ensure complete rehydration. After rehydration, the solutions were heated using a hotplate stirrer. Once the desired temperature was reached, the pH was adjusted using 1 M or 5 M NaOH solutions, depending on the concentration. Once the desired pH was reached, the solutions were kept at the selected temperature and pH conditions for 2 h. Afterwards, the solutions were centrifuged (12,000× *g*, 20 min, 20 °C). The solubilization pellet SP1 was separated from the solubilization supernatant SS1. SP1 was washed with 20 mL of water with the pH adjusted to the same value as the one during the solubilization step. SP1 was vortexed until it was completely dissolved. It was then centrifuged again (12,000× *g*, 20 min, 20 °C) to generate the solubilization pellet SP2 and the solubilization supernatant SS2. SS1 and SS2 were mixed to concentrate all soluble compounds in the same product and form the solubilization supernatant, SS3. SS3 and SP2 were freeze-dried. The obtained powders were then weighted and their protein content analyzed. 

##### Impact of Concentration on Water Lentil Protein Solubilization at Optimized Temperature and pH Values—Second Protocol

Due to the limited number of experiments required for a CCD, the effect of concentration on solubilization yield at the optimal temperature and pH values could not be studied by the protocol described in Section 2.2.1.: First protocol. Therefore, to study this effect, the same steps shown previously (in Section 2.2.1.: First protocol) were repeated until the solubilization supernatant corresponding to SS3 in the previous workflow (Figure 1) was obtained. These experiments were performed in quadruplicate using a full factorial design (FFD). A FFD is an experimental design that allows the estimation of the main effects and interactions of two or more input variables on one or more output variables [47]. The temperature was fixed at 80 °C, the pH at 11, and only the concentration varied between 2–8% *w*/*w*. Then, 10 mL of the supernatant SS3 was collected, freeze-dried, weighted, and its protein content analyzed. 

##### Water Lentil Protein Purification by Isoelectric Point Precipitation—Third Protocol

**Figure 2:**
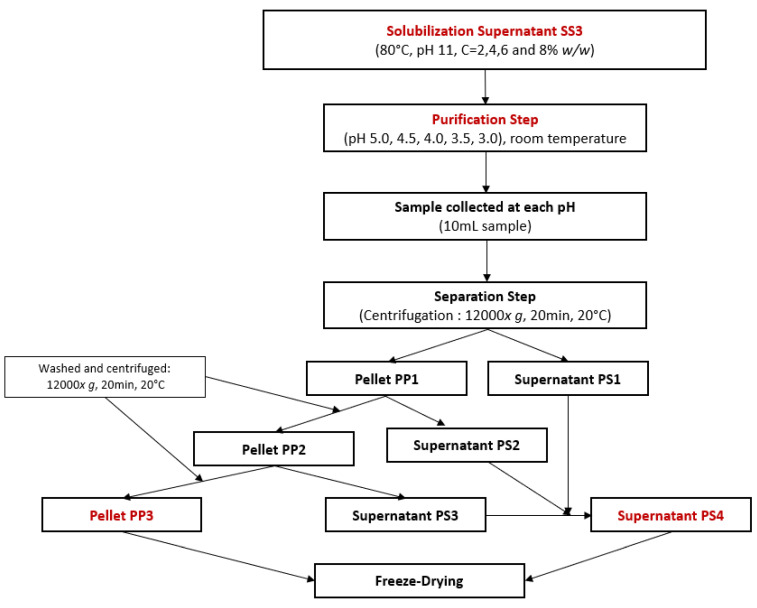
Experimental workflow for protein purification by isoelectric point precipitation using a full factorial design.

For the optimization of protein purification by isoelectric point precipitation, another FFD was used. The objective was to precipitate the previously solubilized proteins and separate them from other soluble elements such as sugars, salts, or soluble fibers. Following the sampling of 10 mL of SS3 in Section 2.2.1: Second protocol, the effect of concentration and pH of purification were tested on the remaining 90 mL of sample at room temperature for total protein yield and protein purity using the experimental workflow presented in Figure 2. The pH was decreased using 6 M, 4 M, 2 M, 1 M, or 0.5 M HCl until it reached pH values of 5.0, 4.5, 4.0, 3.5, and 3.0. At each of these pH values, 10 mL of solution was collected and then centrifuged (12,000× *g*, 20 min, 20 °C). From these samples, the purification pellet PP1 and the purification supernatant PS1 were separated. PP1 was then vortexed in 5 mL of distilled water with pH adjusted to the same value as the one during the precipitation step until PP1 was dissolved. Then, PP1 was centrifuged (12,000× *g*, 20 min, 20 °C) to generate the purification pellet PP2 and the purification supernatant PS2. Then, another 5 mL of distilled water with a pH adjusted to the same value as the one during the precipitation step was added to PP2. The solutions were again vortexed until PP2 was completely dissolved and then centrifuged (12,000× *g*, 20 min, 20 °C). The purification pellet PP3 was separated from the purification supernatant PS3. PS1, PS2, and PS3 were products containing compounds such as salts, sugar, soluble fibers, and proteins that remained soluble despite isoelectric point precipitation. These products were mixed to concentrate all remaining soluble compounds in the same product and form the purification supernatant PS4. Additionally, half of each product was redissolved in distilled water and neutralized at pH 7, which is a common procedure for plant-based protein concentrates and isolates to easily include them in food products [48]. PS4 and PP3 at pH 4 and pH 7 were freeze-dried. 

The obtained powders were then weighted and their protein content analyzed. The protein composition of IP, PS4, and PP3 extracted under the best conditions of purification was studied using proteomic analysis. To further characterize these products, the secondary structure of IP, PS4, and PP3 extracted in the best conditions of purification was studied at pH 4 and pH 7 using ATR-FTIR spectroscopy. Additionally, their quaternary structure at pH 4 and pH 7 was also studied using differential scanning calorimetry. Finally, the solubility and foaming properties of IP, PP3, and PS4 at pH 4 and pH 7 were assessed and compared to egg white as a benchmark. 

#### 2.2.2. Analyses

##### Proximal Composition

Protein content was determined using the Dumas combustion method with a Rapid Micro N Cube (Elementar, Francfort-sur-le-Main, Germany). A nitrogen conversion factor of 5.8 was used based on the research of Nieuwland et al. [13] compared to the usual factor of 6.25. The analysis was carried out in duplicate, and the results were expressed in percentages on a dry basis. Ash content was measured following the AOAC 938.08 method, dietary fiber content by the AOAC 985.29 method, and moisture content by the AOAC 950.46 method. All analyses were carried out in triplicate.

##### Protein Solubilization and Total Protein Yields

The protein solubilization yield corresponded to the mass of protein recovered in SS3 (dry basis) after solubilization in comparison with the mass of protein present in the powder and was calculated according to Equation (1):

(1) $$Solubilization\;Yield=\frac{\%P\;in\;SS3\times m_{SS3}\;}{\%P\;in\;\;IP\times m_{IP}}\;\;\;$$

where %P in SS3 is the percentage of protein in the SS3 powder (after freeze-drying), $m_{SS3}$ the mass of SS3 powder, %P in IP the percentage of protein in the initial powder and $m_{IP}$ the mass of initial powder. 

In Section 2.2.1.: Second protocol, the temperature and pH were fixed at T = 80 °C and pH 11, respectively, and only 10 mL of the total volume of SS3 were collected for this part of the experiment. Therefore, a different protein solubilization yield calculation was needed compared to Section 2.2.1.: First protocol. First, the total volume of SS3 was weighted. Then, 10 mL of SS3 were collected and freeze-dried. After the mass of powder obtained from 10 mL of SS3 was obtained, the liquid/solid ratio (L→S ratio) was calculated using Equation (2):

(2) $$L\to S\;ratio=\frac{{m_{liquid}}_{SS3}}{{m_{solid}}_{SS3}}\;\;\;\;$$

where ${m_{liquid}}_{SS3}$ is the mass of the 10 mL of SS3 liquid solution, and ${m_{solid}}_{SS3}$ , the mass of SS3 after freeze-drying the 10 mL of solution. It was therefore possible to estimate how much powder would have been obtained if the whole volume of liquid SS3 was freeze-dried (Equation (3)).

(3) $${m_{solid}}_{SS3}=L\to S\;ratio\times {m_{tot,\;liquid}}_{SS3}\;\;\;$$

where ${m_{solid}}_{SS3}$ is the mass of solid supernatant S3 powder, L→S ratio  being calculated in Equation (2), ${m_{tot,\;liquid}}_{SS3}\;$ the mass of all the liquid supernatant S3. Knowing the mass of SS3 powder and its protein content, the estimated solubilization yield was calculated using Equation (1).

The total protein yield corresponded to the mass of protein recovered in PP3 (on a dry basis) after solubilization and precipitation in comparison with the mass of protein present in the initial solution. The total protein yield was estimated in the same manner as the solubilization yield when temperature and pH were fixed at 80 °C and pH = 11, respectively, as only 10 mL of solution were collected at each purification pH, using Equations (2) and (3). The total protein yield was then calculated using Equation (4) as follows:

(4) $$Total\;protein\;yield=\frac{\%P\;in\;PP3\times m_{PP3}\;\;}{\%P\;in\;IP\times m_{IP}}$$

where %P in PP3 is the percentage of protein in PP3 powder (after freeze-drying), $m_{PP3}$ the mass of PP3 powder, %P in IP the percentage of protein in the initial powder, $m_{IP}$ the mass of the initial powder. 

##### Protein Identification and Quantification by Proteomic Analyses

Protein digestion

Protein digestion and mass spectrometry experiments were performed by the Proteomics platform of the CHU de Quebec Research Center (Quebec, QC, Canada). The digestion protocol was adapted from Nieuwland et al. [13] with some modifications. Dried samples (IP, PP3, and PS4 produced at an initial powder concentration of 2% and 4% at pH4) were resuspended with 325 uL of S-trap lysis buffer (5% sodium dodecyl sulfate (SDS), 50 mM triethylammoniun bicarbonate (TEAB) pH 8.5, and 20 mM dithiothreitol (DTT) and submitted to bead beating, heated at 95 °C for 10 min, and sonicated with a microprobe (Sonic Dismembrator 550, Fisher Scientific, 20 times 1 s ON/1 s OFF at power intensity 3). After mixing the samples, a volume corresponding to 75 μg of protein was transferred into new tubes. Proteins were reduced with 10 mM tris(2-carboxyethyl)phosphine (TCEP) for 15 min at 55 °C and alkylated with 50 mM iodoacetamide (IAA). Samples were then transferred and digested on S-trap Micro Spin columns using 2 ug of trypsin according to the manufacturer protocol (Protifi, Huntington, NY, USA). Eluted peptides were vacuum dried and resuspended in LC loading solvent (2% ACN, 0.05% trifluoroacetic acid (TFA)). Peptide quantities were estimated using Pierce™ Quantitative Fluorometric Peptide Assay (Thermo Fisher Scientific, Waltham, MA, USA).

Digestion yields, which correspond to the quantity of recovered peptides divided by the quantity of digestible peptides, were calculated using Equation (5):

(5) $$Digestion\;yield=\frac{m_{peptides}}{\left(\frac{{(m}_{sample\;}\times \%P\;in\;sample)}{\;V_{extraction}}\right)\times V_{Strap}}$$

where $m_{peptides}\;$ is the mass of recovered peptides (in mg), $m_{sample\;}$ the mass of dried samples used for digestion (in mg), %P in sample the protein content of the sample, $V_{Strap}$ the volume placed on the S-trap Micro spin (in µL) and $V_{extraction}\;$ the volume of extraction used (400 µL). 

All experiments were performed in duplicate, except for IP and PP3 with an initial powder concentration of 2% at pH 4, which were performed in triplicate.

Mass spectrometry

Mass spectrometry analyses were adapted from Boukil et al. [49]. Approximately 1µg of material was analyzed by nanoLC/MSMS using a Dionex UltiMate 3000 nanoRSLC chromatography system (Thermo Fisher Scientific) connected to an Orbitrap Fusion mass spectrometer (Thermo Fisher Scientific, San Jose, CA, USA) equipped with a nanoelectrospray ion source. Peptides were trapped at 20 μL/min in loading solvent (2% ACN, 0.05% TFA) on a 5 mm × 300 μm C18 pepmap cartridge (Thermo Fisher Scientific) for 5 min. Then, the pre-column was switched online with a 50 cm × 75 µm internal diameter separation column (Pepmap Acclaim column, ThermoFisher), and the peptides were eluted with a linear gradient from 5–40% solvent B (A: 0.1% FA, B: 80% ACN, 0.1% FA) in 30 min at 300 nL/min (60 min total runtime). Mass spectra were acquired using a data dependent acquisition mode using Thermo XCalibur software version 4.1.50. Full-scan mass spectra (350 to 1800 m/z) were acquired in the orbitrap using an AGC target of 4 × 10^5^, a maximum injection time of 50 ms, and a resolution of 120,000. Internal calibration using lock mass on the m/z 445.12003 siloxane ion was used. Each MS scan was followed by the acquisition of fragmentation MSMS spectra of the most intense ions for a total cycle time of 3 s (top speed mode). The selected ions were isolated using the quadrupole analyzer with 1.6 m/z windows and fragmented by Higher energy Collision-induced Dissociation (HCD) with 35% collision energy. The resulting fragments were detected by the linear ion trap at a rapid scan rate with an AGC target of 1e4 and a maximum injection time of 50 ms. Dynamic exclusion of previously fragmented peptides was set for a period of 30 s and a tolerance of 10 ppm.

Database searching

Mascot Generic Format (MFG) peak list files were created using Proteome Discoverer 2.3 software (Thermo). MGF files were then analyzed using Mascot (Matrix Science, London, UK; version 2.5.1). Mascot was set up to search a contaminant database and all NCBI protein entries using “duckweed AND plants” as a keyword (3129 entries), assuming the digestion enzyme trypsin. Mascot was searched with a fragment ion mass tolerance of 0.60 Da and a parent ion tolerance of 10.0 PPM. Carbamidomethyl of cysteine was specified in Mascot as a fixed modification. Deamidation of asparagine and glutamine and oxidation of methionine were specified in Mascot as variable modifications. Two missed cleavages were allowed.

Criteria for protein identification and quantification

Protein identification and quantification were adapted from Boukil et al. [49], Ishihama et al. [50] and Nieuwland et al. [13]. Scaffold (version Scaffold_5.1.2, Proteome Software Inc., Portland, OR, USA) was used to validate MS/MS-based peptide and protein identifications. Peptide identifications were accepted if they could be established with greater than 95.0% probability by the Scaffold Local FDR algorithm. Protein identifications were accepted if they could be established with a greater than 95.0% probability and contained at least two identified peptides. Protein probabilities were assigned by the Protein Prophet algorithm [51]. Proteins that contained similar peptides and could not be differentiated based on MS/MS analysis alone were grouped to satisfy the principles of parsimony [49]. Proteins sharing significant peptide evidence were grouped into clusters. To quantify each identified protein, the emPAI (exponentially modified Protein Abundance Index) display option was used [50]. EmPAI values were normalized with a minimal value of 0.0 for all samples. Protein quantification was achieved by estimating each protein’s weight content in comparison to all identified proteins using Equation (6) [50].

(6) $$Protein\;content\;(weight\;\%)=\frac{emPAI\times MW}{\sum (emPAI\times MW)}\times 100$$

where emPAI is the emPAI value of each identified protein, ∑ (emPAI×MW is the sum of the emPAI values of all identified proteins, each multiplied by their respective molecular weight MW.

Identified proteins were finally grouped into families based on enzyme/protein function, which allowed us to calculate each protein family’s weight proportion compared to all other identified protein families [13].

##### FTIR Spectroscopy

Sample measurement and data acquisition

Protein secondary structure analyses were performed using Attenuated Total Reflection-Fourier Transform Infrared (ATR-FTIR) measurements. The protocol was adapted from Ayala-Bribiesca et al. [52], with slight modifications. The samples (dry powders) were placed in a Nicolet 560 FTIR-Spectrometer (Nicolet Instrument Corp., Madison, WI, USA), covering the complete surface of the zinc selenide ATR crystal (Nicolet Instrument). The spectra were collected using 128 scans at a resolution of 4.0 cm^−1^, with an “ATR” correction, “none” Zero filling, an apodization of “N-B strong,” and a phase correction of "Mertz." The spectra were processed using Omnic 5.1a software (Nicolet Instrument Corp.). The vapor spectrum was subtracted from the sample spectra, followed by a manual baseline correction in the 3800 cm^−1^ to 600 cm^−1^ wavenumber regions. The sample’s spectra were overlayed using the “Common scale” scaling option. Analyses were performed in triplicate. The spectra shown are an average of the triplicate.

Secondary structure analyses using Fourier self-deconvolution on the Amide I band.

Fourier self-deconvolution was performed on the 1800 cm^−1^ to 1500 cm^−1^ region using a bandwidth of 30 and an enhancement of 2.0. The transformed spectra of each sample were overlayed using the “Offset scale” scaling option to normalize the absorbance of each sample, which improved secondary structure qualitative analyses between samples. Several secondary structure components can be attributed to the 1700 cm^−1^ to 1600 cm^−1^ region. It must be noted that this attribution may vary slightly depending on the study and the solution in which the proteins were dispersed [53,54,55]. Indeed, in FTIR spectroscopy, proteins are generally dispersed in H_2_O or D_2_O. However, H_2_O vibrations cause overlaps in the Amide I region, which often leads to its replacement as a solvent by D_2_O [53]. In the present study, freeze-dried powders were studied using FTIR-ATR and were therefore not dispersed in D_2_O or H_2_O. Therefore, a secondary structure attribution based on values established in solutions dispersed in D_2_O was chosen, as there should not be interferences caused by this solvent. Thus, the Amide I band can be divided into intermolecular antiparallel β-sheet (1624–1614 cm^−1^), intramolecular parallel or antiparallel β-sheet (1636–1625 cm^−1^), random coils (1645–1641 cm^−1^), α-helix (1659–1646 cm^−1^) and turns and loops (1674–1662 cm^−1^) [54].

##### Differential Scanning Calorimetry

Thermal analyses were performed with a TA instrument DSC Q1000 (TA Instruments, New Castle, DE, USA), with a nitrogen flow of 50 mL/min and a pressure of around 20 PSI in the nitrogen cylinder. The protocol was adapted from Martin et al. [26] and Calderón-Chiu et al. [56], with some modifications. Indium in a non-hermetic coated aluminum pan was used as calibration, and an empty non-hermetic pan was used as reference. About 2.5–8 mg of samples [56] (containing between 20% and 57% protein) were weighed in non-hermetic pans and then sealed with the appropriate lids using a sample encapsulation press (TA Instruments, New Castle, DE, USA). The temperature scans were performed from 10 °C to 200 °C in linear mode at a rate of 2 °C/min [26]. Data were collected using TA instrument explorer (TA Instrument Inc.) and analyzed using TA Universal Analysis (TA Instrument Inc.). The peak area was determined using the “Integrate Peak Linear” tool. Analyses were performed in triplicate.

##### Functionnal Properties

Solubility

Solubility according to pH was adapted from Mikhaylin et al. [57]. Solutions of 1.5% (*w*/*w*) proteins were prepared by weighing the appropriate mass of sample and distilled water into 2 mL tubes. The solutions were gently stirred overnight at room temperature to ensure complete rehydration. The solutions were then vortexed for 30 s, after which they were photographed, and their solubility was assessed visually.

Foaming Properties

Foams were produced as described by Schwenke et al. [58] with some modifications. The 1.5% (*w*/*w*) protein solutions (2 mL) prepared in Section 2.2.2: Functionnal properties–Solubility were homogenized in a calibrated 10 mL glass cylinder using an Ultra-turrax (T25 basic, IKA-WERKE; Wilmington, NC, USA) at speed 4 (19,000 RPM) for 1 min. The volume of the solution (foam + liquid) and the volume of foam was then recorded directly after homogenization and after 1, 3, 5, 10, 30, 60, and 120 min. The foam capacity was calculated using Equation (7) [59]:

(7) $$Foaming\;capacity\;(FC)=\frac{V_{2\;}-V_{1\;}\;}{V_{1\;}}\times 100$$

where $V_{2}$ (mL) is the volume of protein solution (foam + liquid) immediately after homogenization and $V_{1}$ (mL) is the volume of protein solution (liquid) before homogenization. 

The foaming stability at 1, 3, 5, 10, 30, 60, and 120 min was expressed as the percentage of the foam volume (mL) immediately after homogenization compared to the foam volume (mL) at each time [59]. All analyses were performed in triplicate.

##### Statistical Analyses

Protein solubilization optimization was achieved using a central composite design (CCD), which was produced using JMP^®^ Pro 16 software (SAS Institute Inc., Cary, NC, USA) and adapted from Akyüz and Ersus [60] and Dong et al. [61]. Extraction yield and protein purity (Responses) were set to be maximized, while factors such as temperature, concentration, and pH were set to their minimal and maximal values. The CCD was designed with 2 central points for a total of 16 trials. The axial value was set at 1,0 on the faces. Trials were randomized by the software. Analyses were conducted using a linear model with the standard least squares statistical analysis. The protein solubilization optimization at fixed temperature and pH values and the protein purification optimization were achieved using a full factorial design (FFD). The FFD was generated and analyzed using SAS^®^ Studio available on SAS^®^ OnDemand for Academics. All trials were performed in quadruplicate. A two-way ANOVA using the “proc glm” command was conducted to study the impact of pH and concentration. Statistical differences were analyzed using linear, quadratic, cubic, and residual contrasts, as well as all contrast combinations for both factors [62,63]. Effects were considered significant when the *p*-value was <0.05. Protein quaternary structure (differential scanning calorimetry), foaming capacity, and foaming stability were statistically analyzed using a one-way ANOVA and post-hoc Tukey test with minimal respective *p*-values of 0.05 using the software SigmaPlot (version 12, Systat Software, San Jose, CA, USA). For all analyses and ANOVAs, residual normality and variance homogeneity were confirmed, which meant that no data transformation was needed [64].

## 3. Results and Discussion

### 3.1. Water Lentil Protein Extraction by Solubilization 

Statistical analyses of the linear model using concentration, temperature, pH, their respective interactions, and the quadratic effect of each factor showed a high regression coefficient (R^2^ = 0.94) for the solubilization yield, meaning that 94% of the total observed variability could be explained by the model. Only temperature (*p* = 0.0015), pH (*p* = 0.0007), and their coupled effect “temperature*pH” (*p* = 0.0050) had a significant effect on protein solubilization yield (Table 3).

**Figure 3:**
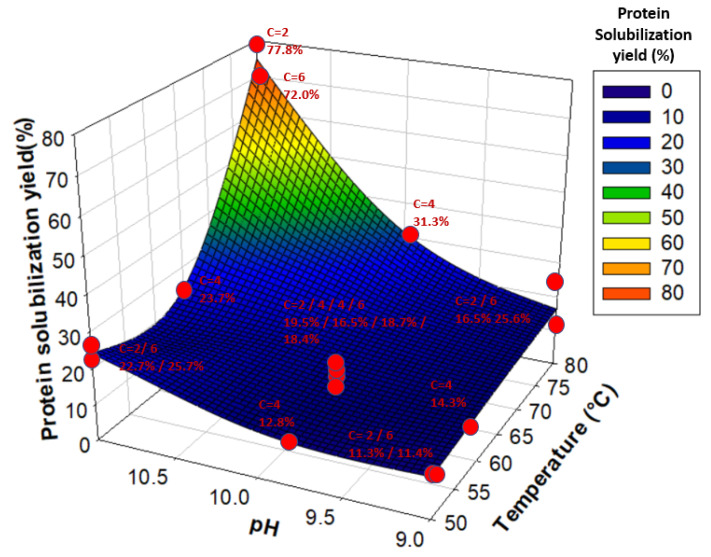
Protein solubilization yield (%) as a function of temperature and pH. Red dots represent each tested sample. “C” stands for Concentration. Yields are associated with their respective Concentration, pH, and Temperature values and are given next to each red dot.

Moreover, the JMPs prediction profiler showed that an increase in temperature without an increase in pH (and vice versa) did not lead to significant improvements in the protein solubilization yield, explaining the coupled effect of Temperature*pH. Therefore, an increase in both temperature and pH values was necessary to maximize protein solubilization, as illustrated in Figure 3. The optimal temperature and pH values for solubilizing water lentil proteins were 80 °C and pH 11, while initial powder concentration did not seem to have any significant effect. Such conditions of temperature and pH led to solubilization yields between 72.0% and 77.8%.

Kobbi et al. [25] found that alfalfa green juice protein solubility was minimal between pH 3 and pH 5 and gradually increased at higher pHs, with a maximal protein solubility of around 90% at pH 10–11. This is due to an increase in the pH of the alkaline region, leading to a higher global charge of the protein compared to the pH values closer to the isoelectric point (pI), leading to more protein-water interactions [65]. In this study, it was observed that an increase in pH was not sufficient to substantially increase water lentil protein solubilization. The solubilization yield only increased by 10 units of percentage between pH 9 and pH 11 for temperatures of 50 °C and 65 °C; however, there was an increase of 55 units of percentage for 80 °C, regardless of the concentration, which questions the role of temperature. Previous studies advocated avoiding high temperatures (80–82 °C) because they could induce aggregation and precipitation of the soluble proteins in the green pellet [66]. In fact, most studies usually use temperatures of around 50 °C to 55 °C to precipitate the green pellet. In the present study, heating at 50 °C or 65 °C did not show any significant improvements in precipitation of the green pellet proteins or solubilization of the white protein fraction. In fact, 75% to 90% of total protein remained in the green pellet at these temperatures, regardless of the concentration or pH values. However, heating at 80 °C at pH 11 allowed solubilization of up to 77.8% of total proteins, with only 11.0% remaining in the green pellet. Indeed, since RubisCO is a large protein composed of 8 small subunits (S) of around 15 kDa and 8 large subunits (L) of around 55 kDa [14], these subunits separate at alkaline pH (>11.0), which could be due to the decrease in hydrophobic interactions between these subunits [14]. Moreover, an increase in temperature could lead to an unfolding of the protein, which could then expose more of its hydrophilic sites to water and induce fewer protein-protein hydrophobic interactions [65]. 

Moreover, Nynäs et al. [21] mentioned that during electrophoresis of leaf protein concentrates, RubisCO bands started to disappear from the white soluble protein fraction at 55 °C and completely disappeared at 65 °C for various leaf sources. They hypothesized that RubisCO proteins became insoluble and precipitated in the green pellet. However, they observed that at 80 °C, a slight RubisCO large L subunit band reappeared, which they attributed to the dissociation of RubisCO L subunits and different solubility behaviors.

Therefore, in our optimal conditions, the hypothesized mechanism would be the following: First, at a high pH value (pH 11) and temperature (80 °C), the separations of the RubisCO subunits were induced. At the same time, these newly dissociated subunits were unfolded because of the high temperature. Such unfolding exposed hydrophilic sites, which could then be charged because of the increase in pH, leading to more protein-water interactions. Such combined effects would explain why the increase of pH and temperature to their maximal values was necessary to maximize protein solubility. Concerning other proteins than RubisCO, such as insoluble chloroplast leaf proteins, similar patterns as RubisCO may have happened, or simply the combined effects of temperature and pH increase induced less protein-protein hydrophobic interactions and more protein-water hydrophilic interactions, which led to an increase in the solubility of these proteins as suggested by Tamayo Tenorio et al. [22]. To the authors’ best knowledge, this is the first time that the positive coupled effect at these temperature and pH values on leaf protein solubilization has been demonstrated. This allowed the solubilization of up to 77.8% of total protein, which doubles the quantity of total proteins contained in the white protein fraction (around 40%) and consisting of soluble proteins only. It was expected that protein concentration could influence protein solubilization; however, such an effect was not observed in the central composite design as the *p*-values associated with the simple or coupled effect of concentration were very high. It is possible that the effects of temperature and pH were so important that they could mask the potential effect of the concentration. 

### 3.2. Impact of Concentration on Water Lentil Protein Solubilization at Optimized Temperature and pH

Following the results of the previous CCD, optimal conditions of temperature and pH for maximizing the solubilization of water lentil proteins were found; however, concentration did not seem to have a significant effect on protein solubilization. It was necessary to verify that concentration did not have any significant effect on solubilization yield at the optimal temperature and pH values. According to the protocol described in Section 2.2.1: Second protocol, a regression model using concentration as the input parameter and protein solubilization yield as the response was generated. The linear, quadratic, and cubic effects of the concentration were tested, and the results are shown in Table 4.

Statistical analyses showed that there was a significant linear effect of concentration on the solubilization yield (*p* = 0.03) associated with the following Equation (8):

(8) Protein solubilization yield=85.35−2.85∗Concentration

meaning that each concentration unit decreased the solubilization yield by 2.85%. These results were in accordance with the previous results in Section 3.1, which demonstrated the repeatability of the experiments and validated the estimation method used to extrapolate the solubilization yields. Such results can be explained by the fact that a higher concentration could lead to an increase in protein-protein hydrophobic interactions, facilitating protein aggregation [67], and thus decreasing protein solubility. 

### 3.3. Water Lentil Protein Purification by Isoelectric Point Precipitation

Following the results presented in Section 3.1 and Section 3.2, the optimal conditions of temperature (80 °C), pH (pH 11), and concentration (2%) were found to maximize water lentil protein extraction by solubilization. However, there was no effect of temperature, pH, or concentration on the protein purity of the obtained product (Table S2), which was very low, around 27.1%, as seen in Figure S3. Therefore, it was necessary to purify the solubilized proteins from other soluble compounds such as salts, sugars, and soluble fibers by isoelectric point precipitation. Thus, this third protocol of purification was carried out with the objectives of maximizing the total protein yield as well as the protein purity of the purification pellet PP. 

#### 3.3.1. Impact of Concentration and pH of Purification on Total Protein Yield

Statistical analysis showed that “pH” had a significant effect (*p* = 0.0007) on protein yield, while “concentration” had no significant effect (*p* = 0.13). In the same way, the interaction “pH*concentration” (*p* = 0.22) had no significant effect on protein yield (Table S3). Further analyses using contrasts showed that the pH of precipitation had a linear (*p* = 0.0064) and a quadratic (*p* = 0.0006) effect on yield and that there was a significant effect of the interaction “Concentration (linear)* pH (linear)” (*p* = 0.035). The effects of concentration for each pH level and the effect of pH for each concentration level on total protein yield were also studied (Table 5).

Hence, the pH of precipitation only had a significant effect on the total protein yield for concentrations of 2% (*p* = 0.0006) and 4% (*p* = 0.0160), and the concentration only had a significant effect on the total protein yield at pH 4 (*p* = 0.021).

Therefore, the optimal conditions for increasing water lentil protein extraction yields by isoelectric point precipitation after the solubilization step are an initial concentration of 2% or 4% with a precipitation pH of 4. With these values, a maximum yield of 60.0% and 57.9%, respectively, of total protein was achieved. It must be noted that other concentration and pH conditions induced a protein yield that was comprised between around 37.8% and 50.3%. Particularly, precipitation at pH 5 and pH 4.5 with an initial concentration of 2% induced a protein yield of 37.8% and 41.0%, respectively.

Nieuwland et al. [13] found that the minimal solubility of their water lentil protein concentrate composed of 92% RubisCO was around pH 5, which suggested that the isoelectric point of water lentil RubisCO was around pH 5. This means that a maximum quantity of RubisCO proteins should have precipitated at pH 5, which would have induced a maximum total protein yield at pH 5. This was not the case in the present study since the maximal total protein yield was obtained at pH 4 for initial powder concentrations of 2% and 4%. The extraction conditions used by Nieuwland et al. [13] likely did not induce the denaturation of RubisCO, as they used sodium metabisulfite, increased the temperature to 52 °C for 30 min, and set the pH at 6, followed by three consecutive centrifugations. Therefore, they likely extracted intact water lentil RubisCO, whose pI may be closer to pH 5. However, the extraction conditions used in the present study may have caused RubisCO denaturation, separating the L subunits from the S subunits due to temperature and alkali solubilization, as suggested in Section 3.1. Moreover, the pI of RubisCO may vary depending on species and RubisCO subunit. Maize leaf complete RubisCO is 4.6, while the pI of three of its large subunits (L) is 5.2, 5.2, and 5.3 and the pI of 2 of its small subunits (S) is 4.5 and 4.7 [68]. The pI of alfalfa RubisCO is 6.0, and between 6.6 and 6.9 for its L subunits and 7.45 for its S subunits [68]. Therefore, the difference in total protein yield according to the pH of precipitation might be due to the pI of the L and S subunits of the water lentil RubisCO, which are still unknown in the literature. These pIs may be closer to pH 4 than to pH 5 for at least one of the subunits. Finally, it is possible that other proteins than RubisCO, such as insoluble chloroplast leaf proteins, were solubilized and thereafter precipitated in the purification step at a higher quantity at pH 4 compared to pH 5 for initial powder concentrations of 2% and 4%, due to their specific pI being closer to pH 4 than pH 5. 

#### 3.3.2. Impact of Concentration and pH of Purification on Total Protein Purity

Concerning protein purity, statistical analyses showed that only “concentration” had a significant effect on protein purity (*p* < 0.0001), while “pH” (*p* = 0.64) and the interaction “concentration*pH” (*p* = 0.52) had no significant effect on protein purity (Table S4). Further analyses with contrasts showed that there was a linear (*p* < 0.001) and cubic effect (*p* = 0.0139) of the concentration on protein purity. The effect of concentration for each pH level and the effect of pH for each concentration level on protein purity were also studied (Table 6). These results confirmed that the pH of precipitation had no significant effect on protein purity regardless of the initial concentration and that the initial concentration had a significant effect on protein purity for the pH values of pH 4.5 (*p* = 0.035) and pH 5 (*p* = 0.00040).

Therefore, the optimal conditions for increasing water lentil protein purity by isoelectric point precipitation after the solubilization step are a precipitation pH of 4.5 or 5.0 with a 2% initial powder concentration. With these values, a maximum protein purity of around 60.1% and 61.1%, respectively, was achieved. It must be noted that other concentration and pH conditions induced a protein purity of around 51.0% to 57.6%. Particularly, precipitation at pH 4 with a 2% and 4% initial powder concentration induced a protein purity of 57.6% and 53.8%, respectively.

These results were surprising because the maximal protein purity was achieved at the pH value that seemed to have the lowest protein yield, as mentioned previously, meaning that fewer proteins precipitated at pH 5; however, they were slightly purer than the ones that precipitated at pH 4. Nynäs et al. [21] performed protein extraction using isoelectric point precipitation on leaves originating from several crops and showed that protein aggregates composed of RubisCO were formed before the theoretical value of the pI of their solutions containing RubisCO and other leaf proteins. In fact, in Nynäs et al. [21] study, RubisCO started to precipitate at values corresponding much more to its theoretical pI value of 5.0 compared to the pI of the solution. Then, by lowering the pH, the solution would reach its theoretical pI, more proteins would precipitate, and higher protein yields would be reached. In the present study, water lentil RubisCO may have started to precipitate as soon as pH 5 was reached, as suggested by Nieuwland et al. [13]. However, the pI of the whole solution may have been closer to pH 4, which would explain why higher yields were reached at pH 4 (60.0% total protein yield compared to 37.8% total protein yield at pH 5 for initial powder concentrations of 2%), as suggested in Section 3.3.1. It is also possible that the proteins, such as membrane proteins, that precipitated at pH 4 were bound to non-protein elements, which would then decrease the protein purity of the solution at this pH even if more proteins precipitated at pH 5 (61.1% protein purity at pH 5 compared to 57.6% for initial powder concentrations of 2%). 

In the present study, it was concluded that the most desirable options were the ones that maximized total protein yield: 60.0% and 57.9% total protein yield with 57.6% and 53.8% protein purity at pH 4 for initial powder concentrations of 2% or 4%, respectively, were preferable to 37.8% and 41.0% total protein yield and 61.1% and 60.1% protein purity at pH 5 and pH 4.5 for initial powder concentrations of 2% and 4%, respectively, since the purities were quite close; however, the protein yield changed by 1.56-fold. These optimized products were called PP pH 4 C2 and PP pH 4 C4 (purification pellets obtained at pH 4 at an initial water lentil powder of 2% or 4%). These protein yields combined with such a high purity are, to the author’s best knowledge, among the highest in the literature for leaf protein extraction and are the highest for leaf protein extraction using isoelectric point precipitation (Table 7). Indeed, in most studies, the white soluble protein fraction is purified by precipitating the proteins using heat coagulation, pH precipitation, or separation methods such as filtration (see the introduction part). This step produces a white leaf protein concentrate consisting of around 70% proteins and containing around 25% of the total leaf proteins and a brown supernatant, which is a by-product and represents 15% of the total leaf proteins [20,21]. Interestingly, the purification supernatant (PS), which is a by-product of the present study’s extraction process, had a protein purity of 20.0% and contained around 16.0% of the total protein. These proteins remained soluble despite the pH shifts leading to isoelectric point precipitation, which could make PS pH 4 C2 and C4 (purification supernatant obtained at pH 4 at an initial water lentil powder of 2% or 4%) products of interest. Indeed, Douillard and Mathan [68] mentioned that when RubisCO subunit dissociation occurred, the S subunits were soluble while the L ones were insoluble in acidic conditions (pH 3.5–5.6). Therefore, as mentioned in Section 3.3.1, the proteins contained in the PS may be one of the RubisCO subunits whose pI was not reached at pH 4, likely the small S subunits.

#### 3.3.3. Protein Identification and Quantification at the Initial and Final Steps of the Process

**Figure 4:**
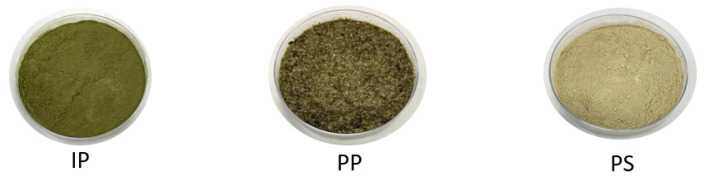
A representative picture of the initial powder (IP), the purification pellet (PP), and the purification supernatant (PS) regardless of pH or concentration.

To confirm the hypotheses made in previous sections concerning the protein composition of the products, proteomics analyses were performed on IP, PP C2 and C4, as well as PS C2 and C4 (Figure 2), which were the best extraction conditions concerning protein yields, and the results are presented in Table 8. The IP protein composition was similar to the leaf protein composition reported in previous studies [13,18,73], with around 45.6% of RubisCO, 2.6% ribosomal proteins, and 51.8% of other insoluble proteins, such as structural proteins (actin), membrane proteins (ATPase, photosystems, CAB protein), or various metabolism proteins. Compared to previous studies, the proportion of RubisCO activase was quite high (18.4%); however, this is likely due to the calculation methodology of the protein abundance index emPAI used in this study. Indeed, this index considers the amino acid coverage of each protein, which was always very high for the RubisCO activase for IP samples, probably since this protein is very small (7 kDa) and its sequence is thus easily covered. PP C2 and C4 were mostly composed of RubisCO (84.5% and 85.6%), but also contained smaller amounts of membrane proteins such as ATPase, CAB protein, or photosystem proteins. The presence of membrane proteins such as CAB protein probably explains the green color of each product, which is presented in Figure 4.

These results are similar to those of studies that isolated RubisCO from leaves, as the final product is usually mostly composed of RubisCO [13]. For instance, Nieuwland et al. [13] successfully produced a LPC from water lentils that contained mostly RubisCO (92%) and ribosomal proteins but almost no membrane proteins (around 2% ATP synthase). This discrepancy with our study is likely due to the differences in the protein’s purification steps between the studies. These results also confirmed the hypothesis suggested in Section 3.3.2 that proteins bound to non-protein elements, such as membrane proteins, precipitated at pH 4, which increased protein yield but slightly decreased protein purity. PS C2 and C4 contained mostly RubisCO (89.6% and 92.1%) and a small fraction of actin, metabolism proteins, and membrane proteins. These results confirmed the other hypothesis suggested in Section 3.2, that RubisCO proteins did not fully precipitate during the purification process. However, the following hypothesis that PS was mostly composed of RubisCO S small subunits that remained soluble in acidic conditions could not be verified, as both PP and PS contained large L and small S RubisCO subunits. Specific analyses using proteomic databases focused on RubisCO proteins would be required to further study this hypothesis. Statistical analyses showed that there was significantly more RubisCO in PP and PS products compared to IP, which indicates that RubisCO was successfully extracted from the IP after the solubilization step, along with a small quantity of other proteins. The purification step led to a fractionation of the RubisCO protein, with around 17.5% total RubisCO remaining in the PS while the rest of RubisCO and other proteins precipitated in the PP. It must be noted that the digestion yields were high for IP (50.5 ± 18.5%) but low for PP C2 and C4 (9.6 ± 1.1% and 10.7 ± 7.4%, respectively) and for PS C2 and C4 (1.5 ± 0.1 and 1.7 ± 0.5, respectively). So, cautious conclusions should be drawn from the results presented in Table 8. However, Nieuwland et al. [13] who used a similar digestion method, did not mention their digestion yields. 

### 3.4. Structural and Functionnal Properties of the Initial and End Products of the Extraction Process

To further characterize the end-products obtained in the optimal purification conditions, protein secondary and quaternary structures using ATR-FTIR spectroscopy and DSC, respectively, were performed at pH 4 and pH 7 on the initial powder (IP) and end products (PP and PS). Moreover, the water solubility and foaming properties of these products were analyzed. The foaming properties of egg white were also measured and compared as a benchmark.

#### 3.4.1. Protein Structural Properties

##### Protein Secondary Structure: ATR-FTIR Spectroscopy

**Figure 5:**
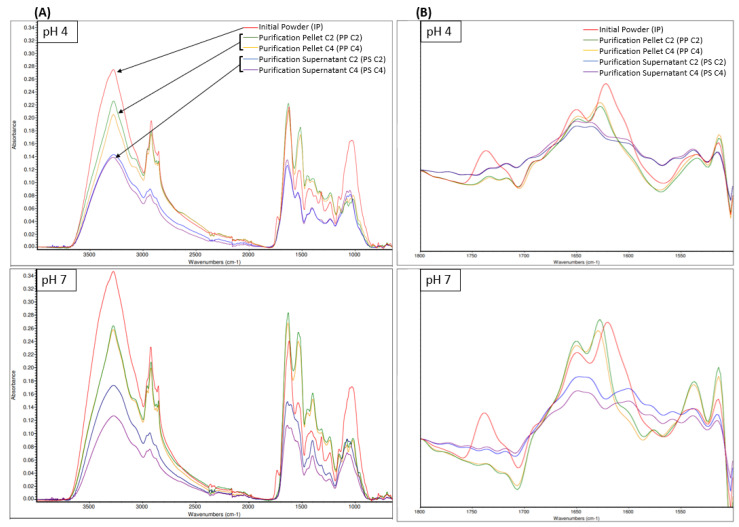
FTIR-ATR spectra of samples at pH 4 and pH 7 of initial powder (IP), purification pellet (PP), and purification supernatant (PS) obtained from an initial powder concentration of 2% and 4% (C2 and C4, respectively) between 4000 cm^−1^ and 600 cm^−1^ (**A**), with a zoom of the Fourier-self-deconvoluted spectra in the 1800 cm^−1^ and 1500 cm^−1^ region (**B**).

The FTIR-ATR spectra (Figure 5) showed similar peaks of absorbance with noticeable differences between the products at the same pH or between the same product at pH 4 or pH 7. In FTIR, several regions can be attributed to the same component. As such, regions 3600 cm^−1^ to 3200 cm^−1^ correspond to an O-H stretching vibration, which is a strong water IR absorption band and can be attributed to water and/or carbohydrates [54]. Considering that our products were freeze-dried, this region will be attributed to carbohydrates only. Regions 3000 cm^−1^ to 2800 cm^−1^ correspond to a C-H stretching of CH_2_ and CH_3_ groups, which can be attributed to the presence of fats, carbohydrates, and proteins. Considering that the initial product was already defatted, this region might be attributed to more carbohydrates and proteins in the present case. Regions 1250 cm^−1^ to 800 cm^−1^ correspond to a C-O stretching and C-O-H bending vibration, which can be attributed to the presence of carbohydrates. For these carbohydrate regions, regardless of the pH, the absorbance was the highest for IP, followed by PP C2 and C4, and then PS C2 and C4. This is likely due to each fraction content in fibers being composed of sugars and containing an O-H chemical bond, with IP having the highest content, followed by PP C2 and C4 and PS C2 and C4.

Moreover, several regions can be attributed to proteins. Indeed, regions 1700 cm^−1^ to 1600 cm^−1^, 1560 cm^−1^ to 1520 cm^−1^ 1300 cm^−1^ to 1190 cm^−1^ correspond to the Amide I, Amide II, and Amide III bands, respectively, which can all be associated with the presence of proteins [54]. For these protein regions, regardless of the pH, PP C2 and C4 had a superior absorbance compared to IP and PS C2 and C4, which can be attributed to the higher protein content of PP products, followed by IP and then PS. For protein analyses using IR spectroscopy, the major region of interest is 1700 cm^−1^ to 1600 cm^−1^ Amide I since the Amide III band can be overlapped by absorptions from other compounds present in the product [54]. Several secondary structure components can be attributed in the 1700 cm^−1^ to 1600 cm^−1^ region. Indeed, the Amide I band can be divided into intermolecular antiparallel β-sheet (1624–1614 cm^−1^), intramolecular parallel or antiparallel β-sheet (1636–1625 cm^−1^), random coils (1645–1641 cm^−1^), α-helix (1659–1646 cm^−1^) and turns and loops (1674–1662 cm^−1^) [54].

To study these secondary structures, Fourier self-deconvoluted spectra of the samples in the 1800 cm^−1^ to 1500 cm^−1^ region are presented in Figure 5, with a focus on the 1750 cm^−1^ to 1600 cm^−1^ region specifically. Interestingly, IP had an absorption peak between 1750 cm^−1^ and 1700 cm^−1^, which corresponds to a C=O stretching vibration and can be assigned to either aspartic acid (Asp) or glutamic acid (Glu) side chains with carboxyl groups [74]. This absorption peak was not present in any PP or PS spectra, meaning that these side chains were either not present or denatured in this sample. Amino acid side chains are often at the core of protein reaction mechanisms [74]. Indeed, Carbonaro et al. [75] established a major positive relationship between amino-acid side chains such as Asp or Glu and protein digestibility. Therefore, the low digestibility observed in Section 3.3.3 for the PP and PS samples may be explained by the absence of Asp or Glu side chains. Regardless of the pH, the IP had an absorbance peak at 1650 cm^−1^ and 1621 cm^−1^. The absorbance peak at 1621 cm^−1^ was higher, which indicates that IP contained a higher proportion of intermolecular antiparallel β-sheet compared to α-helix, which suggests a high protein aggregation [54,76]. This high protein aggregation can be linked to lower protein solubility [76]. Regardless of the pH or concentration, PP also had an absorbance peak at 1649 cm^−1^ and 1628 cm^−1^. The absorption peak at 1628 cm^−1^ was the highest, which indicates that PP contained a higher proportion of intramolecular parallel or antiparallel β-sheet compared to α-helix. However, the peak height and area at 1649 cm^−1^ increased for PP pH 7 compared to PP pH 4 (Figure S4). This means that PP pH 7 contained a higher proportion of α-helix compared to PP pH 4, and α-helix were reported to favor protein solubility [77] since they induce looser protein conformation [54,76]. Regardless of the pH or concentration, PS had an absorbance peak at 1648 cm^−1^ and 1636 cm^−1^, which had similar heights. Therefore, PS seemed to contain a comparable amount of α-helix and intramolecular parallel or antiparallel β-sheet, which may indicate that the PS sample remains soluble at pH 4 and pH 7. 

The FTIR results for the PP products were quite similar to the ones obtained by Calderón-Chiu et al. [56], who analyzed a leaf protein concentrate from jackfruit leaves after solubilization at alkaline pH and a protein precipitation step at pH 4. Using FTIR analyses, they reported 4.49% of α-helix, 56.24% of β-sheet, 38.24% of β-turn, and 1.02% random coil, which somewhat falls in line with our qualitative analyses.

##### Protein Quaternary Structure: Differential Scanning Calorimetry (DSC)

The DSC profiles (Figure S5) of all analyzed samples showed two distinct denaturation peaks, which can be associated with their respective temperatures and peak areas. The peak area corresponds to the enthalpy of transition ∆H per gram of product, and it was normalized according to the protein concentration of the product [78]. This is the enthalpy of transition ∆H per gram of protein. Peak 1 was around 60–74 °C depending on the product, with a high enthalpy of transition values, and peak 2 was around 116 °C to 142 °C, with a very small enthalpy of transition values compared to peak 1 (Table 9).

For peak 1, statistical analyses showed that IP pH 4, IP pH 7, PP pH 4 C2 and C4, PS pH 4 C4, and PS pH 7 C2 and C4 had no significant differences in their denaturation temperatures, which were between 65.4 °C and 74.5 °C. PP pH 7 C2 and C4 and PS pH 4 C2 had the lowest denaturation temperatures, with values between 59.2 °C and 62.9 °C. The denaturation peak 1 may be attributed to different proteins for each product because IP, PP, and PS have different protein compositions (Table 8). However, the observed denaturation temperatures of either IP, PP, or PS fall in line with what is usually observed in RubisCO protein thermal denaturation. In fact, Nieuwland et al. [13] produced a water lentil protein concentrate mostly composed of RubisCO, which showed a denaturation peak at 62 °C at pH 7. Interestingly, PP pH 4 C2 and C4 showed a denaturation peak around 73.5 °C, indicating that these products were not fully denatured at pH 4, which was not observed by Nieuwland et al. [13]. Martin et al. [26] showed that the denaturation temperature of spinach leaf protein concentrate (LPC), which was mainly composed of RubisCO, was around 64.9 °C at pH 7, while Lamsal et al. [33] showed that LPC from alfalfa had a denaturation temperature between 65 °C and 70 °C. These authors did not perform DSC analyses above 120 °C, which may explain why they did not observe a secondary small peak. Concerning the enthalpy of transition ∆H per gram of protein, PS pH 7 C2 and C4 had the highest at around 540 J/g of protein. IP pH 7 had the second highest peak area, around 460 J/g of protein, followed by IP pH 4 and PS pH 4 C2 and C4, which had an enthalpy of transition of around 360 J/g of protein. Finally, whatever the pH or concentration, PP had the same enthalpy of transition between 236.1 and 300.5 J/g of protein. The enthalpy of transition ∆H is the total energy necessary to break noncovalent bonding and thereby denature and unfold a protein [78]. Therefore, a higher enthalpy of transition could indicate that more energy is needed to denature a protein. Thus, PP at pH 4 and pH 7, C2 and C4 proteins required the same amount of energy to be denatured. This was not the case for IP pH 4 and IP pH 7, or PS pH 4 and PS pH 7 C2 and C4, where proteins at pH 7 always required significantly more energy to be denatured. These differences could not be explained by the protein secondary structural changes seen in Section 3.4.1, and in the absence of comparable results in the literature, they remain unexplained. Moreover, a lower denaturation temperature could indicate lower protein stability [13], which has been linked with a lower quantity of hydrogen bonds and polar surface area [79] present in the proteins. Hydrogen bonds confer rigidity to the protein structure and are linked with intramolecular interactions [80]. Therefore, PP pH 7 may contain fewer hydrogen bonds than PP pH 4, and thus fewer intramolecular interactions. This could explain why a higher quantity α-helix and a lower quantity of intramolecular parallel or antiparallel β-sheet were observed for PP pH 7 C2 and C4 compared to PP pH 4 C2 and C4 in the previous section.

For peak 2, statistical analyses showed that IP pH 4, IP pH 7, and both PP pH 4 had the same denaturation temperature at around 138 °C. PS pH 4 and pH 7 C2 and C4 and PP pH 7 C2 and C4 had a very small denaturation peak at around 118.8 °C. IP pH 4 and pH 7 had a higher peak area at 12.8 J/g of protein, followed by PP pH 4 C4 at around 4.7 J/g of protein. All other products had similar denaturation temperatures around 119 °C and similar peak areas, between 0.2 and 3.9 J/g of protein. This secondary peak may indicate some change in non-RubisCO protein quaternary structure, as RubisCO denaturation temperature is well-established. However, to the author’s best knowledge, this small denaturation peak has never been reported in the literature for leaf proteins, which limits its interpretation.

#### 3.4.2. Protein Functionnal Properties according to the Process Step

##### Solubility according to the Process Step

**Figure 6:**
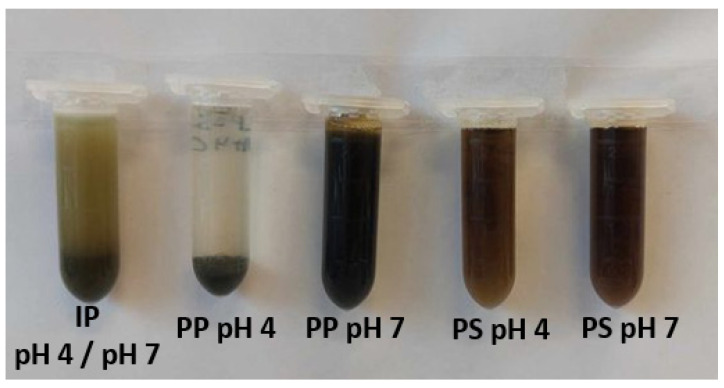
A representative picture of a 1.5% (*w*/*w*) protein solution (2 mL) of the initial powder (IP) at pH 4 and pH 7, the purification pellet (PP) at pH 4 and pH 7, and the purification supernatant (PS) at pH 4 and pH 7 C2 and C4 after being vortexed at full speed for 30 s.

Regardless of the pH, IP was mostly insoluble in water, as seen in Figure 6. The same observation could be made for PP at pH 4, C2, and C4. However, regardless of the concentration values, PP at pH 7 and PS at pH 4 and pH 7 were highly soluble in water. These results were expected for IP as they are composed of around 40.7% insoluble fibers (Table 1) and contain 35.8% proteins, of which 51.8% (Table 8) are insoluble in water [13]. Moreover, these products had high protein aggregation (Section 3.4.1) which can be linked to lower protein solubility [76]. PP pH 4 and pH 7 were mostly composed of RubisCO (Table 8), which was reported to have poor solubility at pH 4 but good solubility at pH 7 [13,80]. Moreover, PP pH 7 products contained more α-helix compared to PP pH 4, which is linked to a looser protein structure [54,76] and was reported to favor protein solubility [77]. To the author’s best knowledge, the solubility of products such as PS was never reported in the literature. However, their solubility at pH 4 and pH 7 can be explained by their high content in α-helix and thus loose protein structure.

##### Foaming Capacity and Stability according to the Process Step

**Figure 7:**
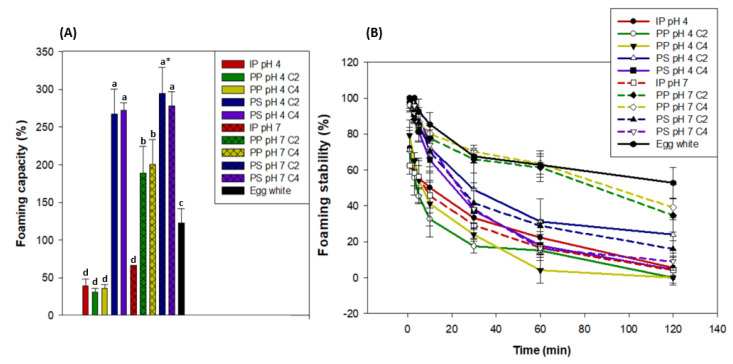
Foaming capacity (**A**) and foaming stability (**B**) of 1.5% protein solution (*w*/*w*) at pH 4 and pH 7 of the initial powder (IP), purification pellet (PP), and purification supernatant (PS) obtained from an initial powder concentration of 2% and 4% (C2 and C4, respectively) compared to egg white (11% protein *w*/*w*). * Significantly different results are marked with letters according to a one-way ANOVA followed by a post-hoc Tukey test (*p* < 0.05). Vertical bars indicate the standard deviation.

Statistical analyses showed that, regardless of the pH or concentration, the PS products had the highest foaming capacity, which was around 278% of the initial volume (2 mL) of the 1.5% protein solution. Regardless of the concentration, PP at pH 7 had the second highest foaming capacity at around 194%, followed by egg white (122%), and then by PP C2 and C4 at pH 4 and IP at pH 4 and pH 7 at around 43% (Figure 7). PS products all had similar protein structure and solubility, and they also contained more salts than other products due to the protein extraction method. Protein structure was shown to influence protein foams, as the less-ordered, looser β-casein formed more foam than the more highly ordered, rigid lysozyme [81]. The proteins contained in the PS contained a large quantity of α-helix as shown in Section 3.4.1, which probably induced a loose protein structure and could thus explain why the PS products had the highest foaming capacity. Moreover, salt content has been reported to improve protein solubility and thus foaming capacity [82] up to a maximal threshold where it starts to cause a salting-out effect, which decreases protein solubility and thus has a depressing effect on foaming capacity [83]. In our case, this maximal salt content threshold may not have been surpassed, which could also explain why PS products had a higher foaming capacity than PP products at pH 7. Furthermore, previous authors showed that leaf protein concentrates (LPCs) from tobacco leaves had similar or superior foaming capacity to egg white [84,85,86]. More recently, Nynäs et al. [87] showed that LPCs could be an alternative to egg white in foams due to their interfacial behavior. Thus, our results fall in line with other studies when comparing PP products at pH 7 and egg white. It must be noted that egg white contains around 10.2% proteins [88] and the water lentil solutions only contained 1.5% proteins, which highlights their remarkable foaming capacity. Finally, PP products at pH 4 and IP at pH 4 and pH 7 had the lowest foaming capacity due to their aggregated structure [81] and low protein solubility [82,83] as seen in Figure 6. 

The foaming stability of all protein solutions decreased over time at different rates, which is an inevitable physical process [89]. The egg white had the highest foaming stability after 120 minutes, as it retained around 52.8% of its initial foam volume. It was followed by PP pH 7 products, which retained around 36.9% of their initial foam volume. The other products all had comparably worse foaming stability, as it did not exceed 23.8% for PS pH 4 C4, for example. However, it must be noted that the PP pH 7, C2, and C4 had a similar foaming stability to egg white up to 60 min, while the foam stability of the other products decreased much faster. The egg white had a higher protein concentration (10.2%) [88], compared to 1.5% for the other products. This may explain why it had a higher foaming stability, as protein concentration can be linked to foam [58,83]. Interestingly, PP pH 7, C2, and C4 had a lower foaming capacity than all PS products but better foaming stability. This duality was also observed by Kinsella [81], as the lysozyme had worse foaming capacity but better foaming stability than the β-casein. Indeed, the lysozyme had more polypeptide interactions due to its highly ordered and rigid conformation, which increased foaming stability. Because of their higher α-helix content (Figure 5), PS products may have an even looser conformation [54,76] with fewer polypeptide interactions than PP pH 7 C2 and C4, which could explain these foaming stability differences. In previous studies, it has been reported that foams were more stable when formed near the isoelectric point than in the alkaline region [39,40]. This is due to lower electrostatic repulsions and thus more compact protein molecules, which form stronger and more stable films [33,40]. Such results were not observed in the present study, as PP pH 4, C2, and C4 had bad foam stability compared to these products at pH 7. Indeed, PP products at pH 4 and IP at pH 4 and pH 7 had the lowest foaming stability, which is linked to their low protein solubility [82,83].

## 4. Conclusions

In this study, we developed a simple and optimized purification process that allowed the extraction of a high quantity of water lentil proteins (60.0% yield) combined with a high protein purity (57.6% purity) at lab scale. The water lentil protein concentrate and its byproduct were mainly composed of RubisCO. The protein concentrate was insoluble in water at pH 4 but soluble at pH 7 due to protein structural changes observed by FTIR and DSC. Moreover, at pH 7, the foaming capacity of a 1.5% protein solution of the water lentil protein concentrate (194%) was superior to a 10.2% protein egg white solution (122%) by a 1.59-fold, while its foaming stability was slightly worse after 120 min. Furthermore, the by-product of the protein concentrate contains some proteins and a significant quantity of salts and may be more valuable if it is demineralized using processes such as electrodialysis. These results confirm that water lentils could be a viable source of protein for human nutrition and food formulation. To confirm the viability of this process, more techno-functional properties such as gelling or emulsifying properties and digestibility should be tested with scaled-up productions.

## Figures and Tables

**Table 1:** Proximal composition of water lentil initial powder (IP) expressed in g/100 g of dry basis and comparison with data reported in the literature.

Food Component	Amount (g/100 g) on Dry Basis				
	Present Study	Rusoff et al. [11]	Yu et al. [44]	Nieuwland et al. [13]	Duangjarus et al. [45]
Crude protein	35.8 ± 0.3	25.2–36.4	34.5	33.6 ± 0.9	33.2 ± 1.9
Fat	-	4.5–6.6	3.9	3.4 ± 0.2	3.0 ± 0.2
Ash	6.2 ± 0.2	13.7–17.1	9.7	18.0 ± 0.4	14.6 ± 0.1
Total carbohydrates	-	-	-	3.3 ± 0.2	36.7 ± 2.0
Dietary fibers	47.2 ± 1.2	8.8–11.0	10.2	25.5 ± 0.7	12.5 ± 0.2
soluble fibers	6.5 ± 0.2	-	-	-	-
insoluble fibers	40.7 ± 1.0	-	-	-	-
Humidity	3.7 ± 0.3	-	-	-	8.6 ± 0.0

Means ± standard deviation.

**Table 2:** Central composite design for water lentil protein extraction by solubilization.

Trial	Input Parameters		
	Concentration (*w*/*w* in %)	Temperature (°C)	pH
1	2	80	9
2	4	80	10
3	4	65	9
4	4	65	10
5	6	80	9
6	6	80	11
7	6	65	10
8	6	50	9
9	2	50	11
10	4	50	10
11	4	65	10
12	2	80	11
13	2	50	9
14	4	65	11
15	6	50	11
16	2	65	10

**Table 3:** Analyses of variance for the protein solubilization yields.

Input Parameter	DF	SS	MS	F-Value	*p*-Value
**Model**	9	5663.85	629.32	11.30	**0.0040**
**Concentration**	1	1.44	1.44	0.03	0.88
**Temperature**	1	1690.78	1690.78	30.36	**0.0015**
**pH**	1	2303.11	2303.11	41.36	**0.0007**
Concentration*Temperature	1	9.81	9.81	0.18	0.69
Concentration*pH	1	0.99	0.99	0.018	0.90
Temperature*pH	1	1041.50	1041.50	18.70	**0.0050**
Concentration*Concentration	1	47.40	47.40	0.85	0.39
Temperature*Temperature	1	140.70	140.70	2.53	0.16
pH*pH	1	47.85	47.85	0.86	0.39
Error	6	334.18	55.69		
Total	15	5997.96			

DF: Degree of freedom; SS: Sum of squares; MS: Mean of squares. In bold the value is statistically significant at *p* < 0.05. In bold; statistically significant effect at *p*-Value < 0.05 level.

**Table 4:** Analysis of variance of the regression model studying the effect of initial powder concentration on the solubilization yield at T = 80 °C and pH 11.

Input Parameter	DF	SS	MS	F-Value	*p*-Value
**Model**	3	679.87	226.62	4.17	**0.031**
**Concentration**	3	679.87	226.62	4.17	**0.031**
**Concentration (linear)**	1	651.51	651.51	11.99	**0.004**
Concentration (quadratic)	1	24.30	24.55	0.45	0.52
Concentration (cubic)	1	4.10	4.09	0.08	0.79
Error	12	652.16	54.35		
Total	15	1332.02			

DF: Degree of freedom; SS: Sum of squares; MS: Mean of squares. In bold; statistically significant effect at *p*-Value < 0.05 level.

**Table 5:** Effect of the pH of precipitation on protein yield for each level of concentration and effect of initial powder concentration on protein yield for each level of pH of precipitation.

Input Parameter	DF	SS	MS	F-Value	*p*-Value
**pH effect at Concentration = 2%**	4	1192.26	298.06	5.74	**0.0006**
**pH effect at Concentration = 4%**	4	689.22	172.3	3.32	**0.016**
pH effect at Concentration = 6%	4	106.5	26.63	0.51	0.73
pH effect at Concentration = 8%	4	19.39	4.85	0.09	0.98
Concentration effect at pH 3	3	95.13	31.71	0.61	0.61
Concentration effect at pH 3.5	3	161.22	53.74	1.04	0.38
**Concentration effect at pH 4**	3	545.1	181.7	3.50	**0.021**
Concentration effect at pH 4.5	3	146.27	48.76	0.94	0.43
Concentration effect at pH 5	3	200.68	66.89	1.29	0.29

DF: Degree of freedom; SS: Sum of squares; MS: Mean of squares. In bold the value is statistically significant at *p* < 0.05. In bold; statistically significant effect at *p*-Value < 0.05 level.

**Table 6:** Effect of the pH of precipitation on protein purity for each level of concentration and effect of initial powder concentration on protein purity for each level of pH of precipitation.

Input Parameter	DF	SS	MS	F-Value	*p*-Value
pH effect at Concentration = 2%	4	147.71	36.92	1.97	0.11
pH effect at Concentration = 4%	4	76.94	19.26	1.03	0.40
pH effect at Concentration = 6%	4	12.44	3.11	0.17	0.95
pH effect at Concentration = 8%	4	19.13	4.78	0.26	0.91
Concentration effect at pH 3	3	95.9	31.97	1.71	0.17
Concentration effect at pH 3.5	3	11.10	3.70	0.20	0.90
Concentration effect at pH 4	3	62.08	20.70	1.11	0.35
**Concentration effect at pH 4.5**	3	171.97	57.32	3.07	**0.035**
**Concentration effect at pH 5**	3	392.69	130.90	7.00	**0.00040**

DF: Degree of freedom; SS: Sum of squares; MS: Mean of squares. In bold the value is statistically significant at *p* < 0.05. In bold; statistically significant effect at *p*-Value < 0.05 level.

**Table 7:** Total protein yield (%) and protein purity (%) of water lentil protein concentrate and of leaf protein concentrate reported in the literature obtained on various plant leaf materials using isoelectric point precipitation.

Leaf Source	Purification pH	Total Protein Yield (%)	Protein Purity (%)	Reference
Alfalfa	4	8.4%	73.9%	[24]
Alfalfa	3.5	36%	60%	[36]
Alfalfa	3	NR	73.0%	[25]
Alfalfa	3.5	NR	72%	[33]
Alfalfa	4.5	29.6%	52.9%	[69]
Alfalfa	4.5	1.5%	12.9%	[21]
Alfalfa	4	Nr.	86,5%	[38]
Alfalfa	4	6%	41%	[70]
Beetroot	4.5	3.8%	7.21%	[21]
Broccoli	4.5	2.9%	7.9%	[21]
Cabbage	4.5	0.2%	0.7%	[21]
Carrot	4.5	0.2%	1.1%	[21]
Cassava	4	48.7%	50.0%	[28]
Cauliflower	4	1.2%	53.1%	[71]
Eastern cottonwood	4	~7.8%	~59.5%	[31]
Lebeeck tree	4	6.0%	37.3%	[32]
Kale	4.5	2.1%	7.9%	[21]
Mangold	4.5	3.7%	7.2%	[21]
Moringa	4–4.5	7.3%	26.9%	[27]
Red clover	4	5.9%	45.3%	[70]
Ryegrass	4	5.1%	61.7%	[70]
Sea lettuce	2	5%	30%	[72]
Sugar beet	3	NR	63.2–82.2%	[37]
Sugar beet	4.5	32.7%	~59%	[34]
Sugar beet	4.5	0.9%	7.2%	[21]
Tall fescus	4	5.2%	60.7%	[70]
White clover	4	3.9%	46.9%	[70]
Water lentil	3.5	NR	NR	[45]
Water lentil	6	14.2%	67.2%	[13]
Water lentil	3.65	NR	44.7%	[11]
Water lentil	3.65	52.1% (1) 45.6% (2) 44.3% (3)	46.1% (1) 67.83% (2) 45.20% (3)	[44]
Water lentil	4	60%	57%	Present study

NR: Not Reported in this study.

**Table 8:** Protein composition in the protein content of samples of initial powder (IP), purification pellet (PP), and purification supernatant (PS) obtained from an initial powder concentration of 2% and 4% (C2 and C4, respectively).

Protein Type	IP	PP C2	PP C4	PS C2	PS C4
Actin	0.9 ± 0.4 ^a,b,^*	0.2 ± 0.3 ^b^	0.1 ± 0.1 ^b^	2.2 ± 1.0 ^a^	2.5 ± 1.3 ^a^
ATPase	14.1 ± 2.0 ^a^	4.7 ± 6.5 ^a,b^	1.8 ± 0.4 ^b^	1.1 ± 0.9 ^b^	0.0 ± 0.0 ^b^
Cytochrome	0.8 ± 0.2 ^a^	0.7 ± 0.6 ^a^	0.9 ± 1.1 ^a^	1.9 ± 1.0 ^a^	0.7 ± 1.1 ^a^
CAB protein	2.7 ± 0.4 ^a^	1.9 ± 1.4 ^a^	3.1 ± 3.0 ^a^	1.3 ± 1.7 ^a^	0.0 ± 0.0 ^a^
Histone	0.0 ± 0.0 ^a^	0.2 ± 0.2 ^a^	0.4 ± 0.5 ^a^	0.0 ± 0.0 ^a^	0.0 ± 0.0 ^a^
Metabolism	8.5 ± 1.7 ^a^	2.1 ± 1.3 ^b^	1.8 ± 1.0 ^b^	2.7 ± 1.3 ^b^	4.2 ± 1.0 ^a,b^
Photosystem	6.1 ± 2.2 ^a^	3.6 ± 3.3 ^a^	4.5 ± 5.1 ^a^	1.1 ± 0.8 ^a^	0.4 ± 0.6 ^a^
Ribosomal	2.6 ± 0.9 ^a^	1.6 ± 1.1 ^a^	1.6 ± 1.6 ^a^	0.0 ± 0.0 ^a^	0.0 ± 0.0 ^a^
RubisCO	45.6 ± 6.1 ^b^	84.5 ± 14.9 ^a^	85.6 ± 12.8 ^a^	89.6 ± 7.0 ^a^	92.1 ± 0.7 ^a^
RubisCO activase	18.4 ± 12.4 ^a^	0.4 ± 0.5 ^b^	0.2 ± 0.1 ^b^	0.1 ± 0.2 ^b^	0.0 ± 0.0 ^b^

* Significantly different results are marked with letters according to a one-way ANOVA followed by a post-hoc Tukey test (*p* < 0.05). Means ± standard deviation.

**Table 9:** Denaturation temperature and enthalpy of transition ΔH in J/g and in J/g of protein of the sample at pH 4 and pH 7 of initial powder (IP), purification pellet (PP), and purification supernatant (PS) obtained from an initial powder concentration of 2% and 4% (C2 and C4, respectively) using differential scanning calorimetry.

Sample	PEAK 1			PEAK 2		
	Denaturation Temperature (°C)	Enthalpy of Transition ΔH (J/g of Product)	Enthalpy of Transition ΔH (J/g of Protein)	Denaturation Temperature (°C)	Enthalpy of Transition ΔH (J/g of Product)	Enthalpy of Transition ΔH (J/g of Protein)
IP pH 4	72.4 ± 1.9 ^a,^*	134.6 ± 5.0 ^c,d^	374.0 ± 13.8 ^c^	134.2 ± 2.6 ^a^	5.0 ± 0.1 ^a^	13.8 ± 0.2 ^a^
PP pH 4 C2	72.2 ± 4.1 ^a^	139.2 ± 18.4 ^b,c^	241.4 ± 43.1 ^e^	138.5 ± 2.7 ^a^	2.0 ± 1.3 ^b,c^	3.4 ± 2.4 ^b,c^
PP pH 4 C4	74.8 ± 2.6 ^a^	127.8 ± 14.2 ^c,d^	236.1 ± 17.5 ^e^	142.2 ± 0.1 ^a^	2.5 ± 1.0 ^b^	4.7 ± 2.3 ^b^
PS pH 4 C2	59.2 ± 6.5 ^c^	69.9 ± 4.0 ^e^	349.3 ± 19.9 ^c,d,e^	116.0 ± 1.6 ^b^	0.8 ± 0.2 ^c,d^	3.9 ± 1.0 ^b,c^
PS pH 4 C4	65.8 ± 3.1 ^a,b^	73.0 ± 6.9 ^e^	364.9 ± 34.5 ^c,d^	118.9 ± 6.4 ^b^	0.3 ± 0.3 ^d^	1.2 ± 1.4 ^c^
IP pH 7	73.7 ± 2.6 ^a^	166.8 ± 4.7 ^a^	463.4 ± 13.2 ^b^	137.0 ± 2.9 ^a^	4.3 ± 0.3 ^a^	11.8 ± 0.8 ^a^
PP pH 7 C2	62.9 ± 0.4 ^b^	167.0 ± 11.0 ^a^	288.0 ± 9.0 ^e^	117.5 ± 0.8 ^b^	0.1 ± 0.1 ^d^	0.2 ± 0.1 ^c^
PP pH 7 C4	62.0 ± 0.6 ^b^	162.3 ± 11.3 ^a,b^	300.5 ± 21.6 ^d,e^	118.6 ± 1.9 ^b^	0.5 ± 0.4 ^c,d^	0.9 ± 0.1 ^c^
PS pH 7 C2	65.6 ± 3.3 ^a^	109.2 ± 5.1 ^d^	545.8 ± 25.7 ^a^	123.1 ± 0.2 ^b^	0.2 ± 0.0 ^d^	0.9 ± 0.1 ^c^
PS pH 7 C4	65.4 ± 3.4 ^a^	106.7 ± 10.5 ^d^	533.6 ± 52.3 ^a^	118.7 ± 5.7 ^b^	0.2 ± 0.2 ^d^	0.8 ± 1.0 ^c^

* Significantly different results are marked with letters according to a one-way ANOVA followed by a post-hoc Tukey test (*p* < 0.05). Means ± standard deviation.

## Data Availability

The data presented in this study are available on request from the corresponding author.

## Supplementary Materials

The following supporting information can be downloaded at: https://www.mdpi.com/2304-8158/12/18/3424/s1. Figure S1: Protein solubilization yield (%) as a function of temperature and pH. Red dots represent each tested sample. “C” stands for Concentration for the preliminary tests. Yields are associated with their respective Concentration, pH, and Temperature values and are given next to each red dot. Figure S2: Protein purity as a function of temperature and pH. Red dots represent each tested sample. “C” stands for Concentration for the preliminary tests. Yields are associated with their respective Concentration, pH, and Temperature values and are given next to each red dot. Figure S3: Protein purity as a function of temperature and pH. Red dots represent each tested sample. “C” stands for Concentration for the preliminary tests. Yields are associated with their respective Concentration, pH, and Temperature values and are given next to each red dot. Figure S4: Fourier self-deconvoluted FTIR-ATR spectra zoomed in the 1800 cm^−1^ and 1500 cm^−1^ regions of the sample at pH 4 and pH 7 of purification pellet (PP) and purification supernatant (PS) obtained from an initial powder concentration of 2% and 4% (C2 and C4, respectively); Figure S5: Representative Differential Scanning Calorimetry profiles of samples at pH 4 and pH 7 of the initial powder (IP), purification pellet (PP), and purification supernatant (PS) obtained from an initial powder concentration of 2% (C2) The curve areas were not normalized by protein concentration. Table S1: Preliminary central composite design for water lentil extraction by solubilization; Table S2: Analysis of variance for the protein solubilization purity; Table S3: Analysis of variance of the full factorial design studying the effect of initial powder concentration and pH of precipitation on the total protein yield; Table S4: Analysis of variance of the full factorial design studying the effect of initial powder concentration and pH of precipitation on the total protein purity.
