# Correction: Muller et al. Optimization of Water Lentil (Duckweed) Leaf Protein Purification: Identification, Structure, and Foaming Properties. *Foods* 2023, *12*, 3424

**DOI:** 10.3390/foods13081218

**Published:** 2024-04-17

**Authors:** Tristan Muller, Marie-Ève Bernier, Laurent Bazinet

**Affiliations:** 1 Institute of Nutrition and Functional Foods (INAF), Department of Food Sciences, Université Laval, Quebec, QC G1V 0A6, Canada; tristan.muller.1@ulaval.ca (T.M.); marie-eve.bernier.7@ulaval.ca (M.-È.B.); 2 Laboratoire de Transformation Alimentaire et Procédés Électro Membranaires (LTAPEM), Laboratory of Food Processing and Electro Membrane Processes, Université Laval, Quebec, QC G1V 0A6, Canada

In the original publication [1], there was a mistake in Table 1: Proximal composition of water lentil initial powder (IP) expressed in g/100 g of dry basis and comparison with data reported in the literature, as published. The error lies in the numerical values in the second column labelled “Present Study”. Upon closer examination, it was evident that the values for soluble fibers and insoluble fibers were inadvertently reversed. The correct values are as follows: Dietary fibers: 47.2 ± 1.2; Soluble fibers: 6.5 ± 0.2; Insoluble fibers: 40.7 ± 1.0. The authors state that the scientific conclusions are unaffected. This correction was approved by the Academic Editor. The original publication has also been updated.
