# Supplementary material for: Optimization of Water Lentil (Duckweed) Leaf Protein Purification: Identification, Structure, and Foaming Properties
Source: Foods. 2023 Sep 14;12(18):3424. doi: 10.3390/foods12183424 (PMC10529404; doi:10.3390/foods12183424)
Supplement: Supplementary file 1 [file foods-12-03424-s001.zip › foods-2601625-supplementary.pdf]

Supplementary material

Table S1: Preliminary central Composite design for water lentil extraction by solubilization

**Trial**	**Input parameters**		
	**Concentration (*w*/*w* in %)**	**Temperature (°C)**	**pH**
1	4	80	8
2	4	50	9.5
3	6	50	9.5
4	6	50	11
5	6	80	9.5
6	4	20	8
7	6	50	9.5
8	8	80	11
9	4	80	11
10	8	20	8
11	8	80	8
12	8	20	11
13	6	20	9.5
14	4	20	11
15	8	50	9.5
16	6	50	8

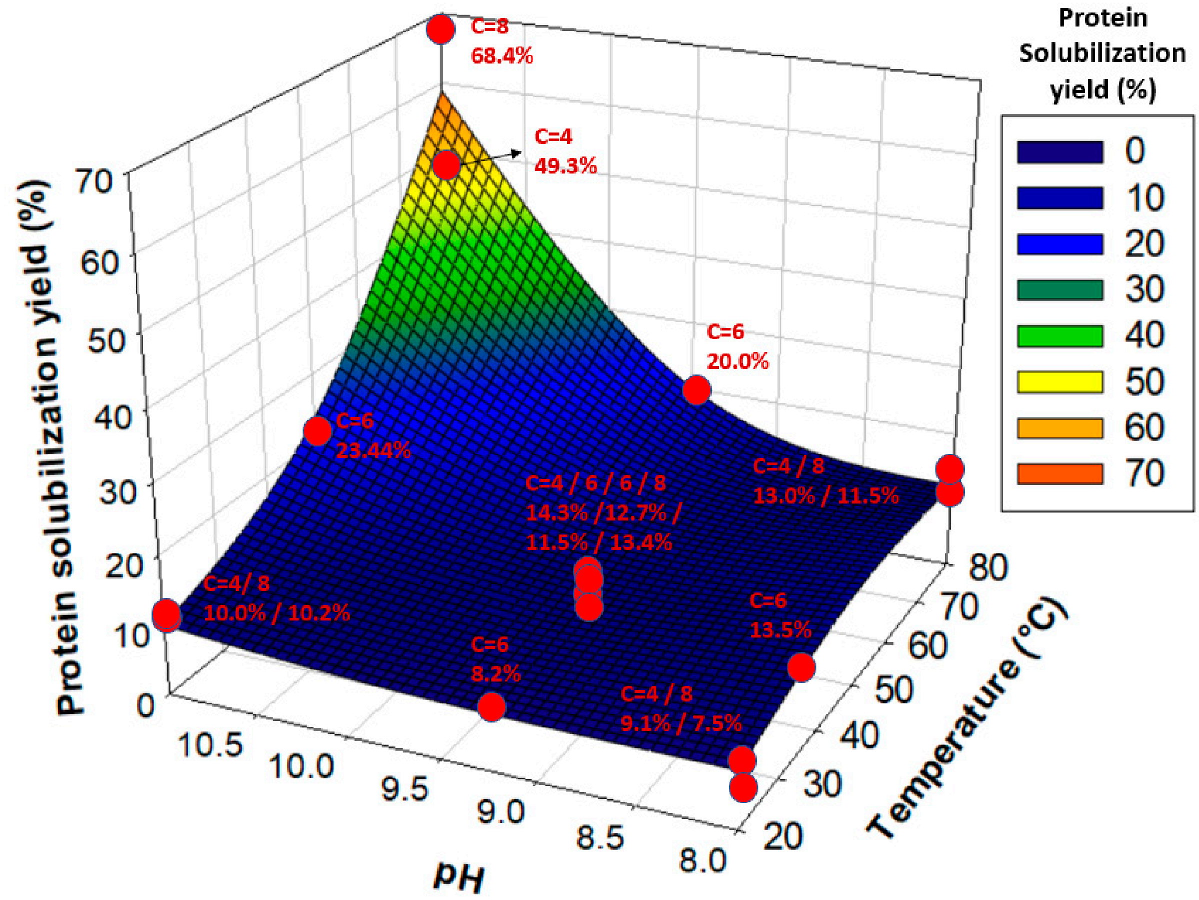

Figure S1: Protein solubilization yield (%) as a function of temperature and pH. Red dots represent each tested sample. "C" stands for Concentration for the preliminary tests. Yields are associated with their respective Concentration, pH and Temperature values and are given next to each red dot.

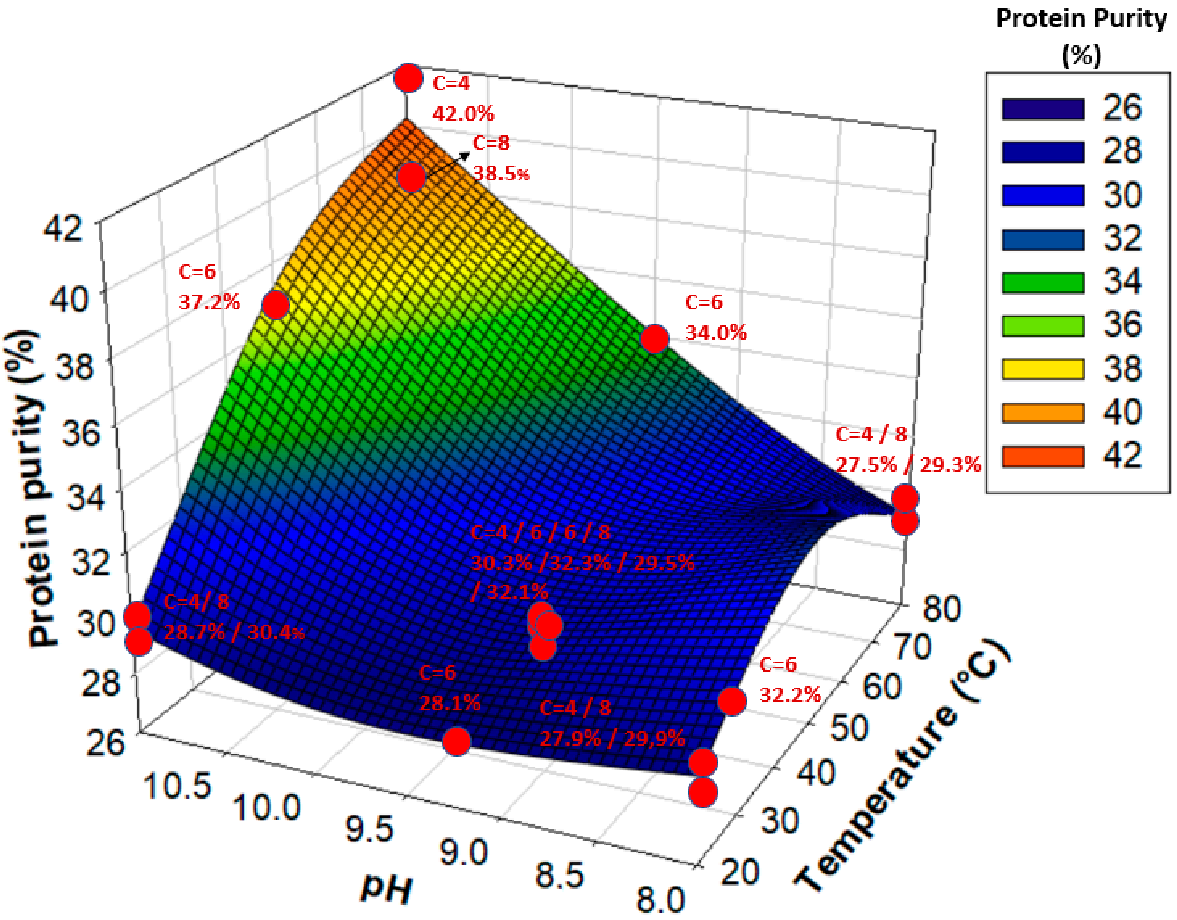

Figure S2: Protein purity as a function of temperature and pH. Red dots represent each tested sample. “C” stands for Concentration for the preliminary tests. Yields are associated with their respective Concentration, pH and Temperature values and are given next to each red dot.

Table S2: Analyses of variance for the protein solubilization purity

Input parameter	DF	SS	MS	F-value	P-value
Model	9	154.1	17.1	0.85	0.60
Concentration	1	65.79	65.79	3.28	0.12
Temperature	1	20.36	20.36	1.01	0.35
pH	1	2.61	2.61	0.13	0.73
Concentration*Temperature	1	5.28	5.28	0.26	0.63
Concentration*pH	1	16.19	16.19	0.81	0.40
Temperature*pH	1	0.096	0.096	0.0048	0.95
Concentration*Concentration	1	17.29	17.29	0.86	0.39
Temperature*Temperature	1	1.12	1.12	0.056	0.056
pH*pH	1	1.78	1.78	0.089	0.78
Error	6	120.38	20.06		
Total	15	274.48			

DF: Degree of freedom; SS: Sum of squares; MS: Mean of squares

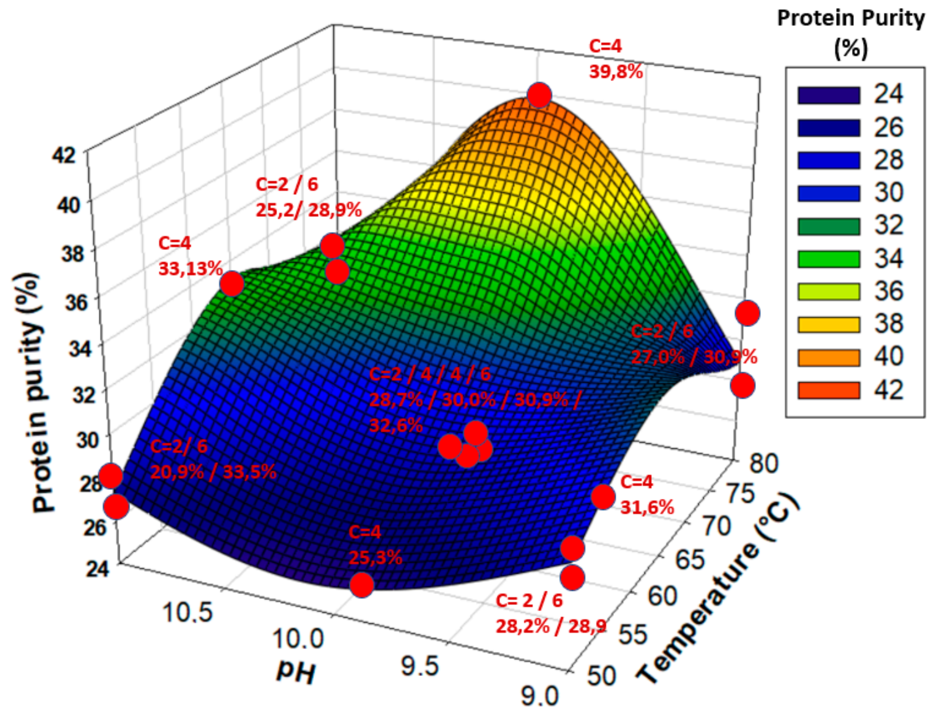

Figure S3: Protein purity as a function of temperature and pH. Red dots represent each tested sample. "C" stands for Concentration for the preliminary tests. Yields are associated with their respective Concentration, pH and Temperature values and are given next to each red dot.

Table S3: Analysis of variance of the full factorial design studying the effect of initial powder concentration and pH of precipitation on the total protein yield.

Input parameter	DF	SS	MS	F-value	P-value
**Model**	19	2315.00	121.84	2.35	**0.0064**
Concentration	3	307.63	102.54	1.98	0.13
Concentration (linear)	1	138.06	138.06	2.66	0.1082
Concentration (quadratic)	1	51.84	51.84	1.00	0.3216
Concentration (cubic)	1	117.72	117.72	2.27	0.1373
**pH**	4	1166.60	291.65	5.62	**0.0007**
**pH (linear)**	1	414.74	414.74	7.99	**0.0064**
**pH (quadratic)**	1	683.90	683.90	13.17	**0.0006**
pH (cubic)	1	0.24	0.24	0.00	0.9460
pH (residual)	1	67.71	67.71	1.30	0.26
Concentration * pH	12	840.77	70.06	1.35	0.22
**Concentration (linear) * pH (linear)**	1	242.44	242.44	4.67	**0.035**
Concentration (linear) * pH (quadratic)	1	188.11	188.11	3.62	0.062
Concentration (linear) * pH (cubic)	1	15.34	15.34	0.30	0.59
Concentration (linear) * pH (residual)	1	170.80	170.80	3.62	0.0618
Concentration (quadratic) * pH (linear)	1	11.56	11.56	0.22	0.64
Concentration (quadratic) * pH (quadratic)	1	0.00	0.00	0.00	1.00
Concentration (quadratic) * pH (residual)	1	141.15	141.15	2.72	0.10
Concentration (cubic) * pH (linear)	1	40.59	40.59	0.78	0.38
Concentration (cubic) * pH (quadratic)	1	2.80	2.80	0.05	0.82
Concentration (cubic) * pH (cubic)	1	3.73	3.73	0.07	0.79
Concentration (cubic) * pH (residual)	1	7.36	7.36	0.14	0.71
Error	60	3114.59	51.91		
Total	79	5429.59			

DF: Degree of freedom; SS: Sum of squares; MS: Mean of squares

In bold ; statistically significant effect at p-Value < 0.05 level

Table S4: Analysis of variance of the full factorial design studying the effect of initial powder concentration and pH of precipitation on the total protein purity.

Input parameter	DF	SS	MS	F-value	P-value
**Model**	19	780.70	41.09	2.20	**0.011**
**Concentration**	3	524.47	174.82	9.35	**<0.00010**
**Concentration (linear)**	1	369.22	369.22	19.74	**<0.00010**
Concentration (quadratic)	1	35.25	35.25	1.88	0.1749
**Concentration (cubic)**	1	120.01	120.01	6.42	**0.014**
pH	4	46.96	11.74	0.63	0.64
pH (linear)	1	0.085	0.085	0.00	0.95
pH (quadratic)	1	13.36	13.36	0.71	0.40
pH (cubic)	1	32.49	32.49	1.74	0.19
pH (residual)	1	1.02	1.02	0.05	0.82
Concentration * pH	12	209.26	17.44	0.93	0.52
Concentration (linear) * pH (linear)	1	40.91	40.91	2.19	0.14
Concentration (linear) * pH (quadratic)	1	22.26	22.26	1.19	0.28
Concentration (linear) * pH (cubic)	1	14.93	14.93	0.80	0.38
Concentration (linear) * pH (residual)	1	1.80	1.80	0.10	0.76
Concentration (quadratic) * pH (linear)	1	42.54	45.24	2.27	0.14
Concentration (quadratic) * pH (quadratic)	1	23.86	23.86	1.28	0.26
Concentration (quadratic) * pH (residual)	1	3.44	3.44	0.18	0.67
Concentration (cubic) * pH (linear)	1	30.93	30.93	1.65	0.20
Concentration (cubic) * pH (quadratic)	1	21.8	21.8	1.17	0.28
Concentration (cubic) * pH (cubic)	1	3.39	3.39	0.18	0.67
Concentration (cubic) * pH (residual)	1	1.48	1.48	0.08	0.78
Error	60	1122.03	41.09		
Total	79	1902.7			

DF: Degree of freedom; SS: Sum of squares; MS: Mean of squares

In bold ; statistically significant effect at *p*-Value < 0.05 level

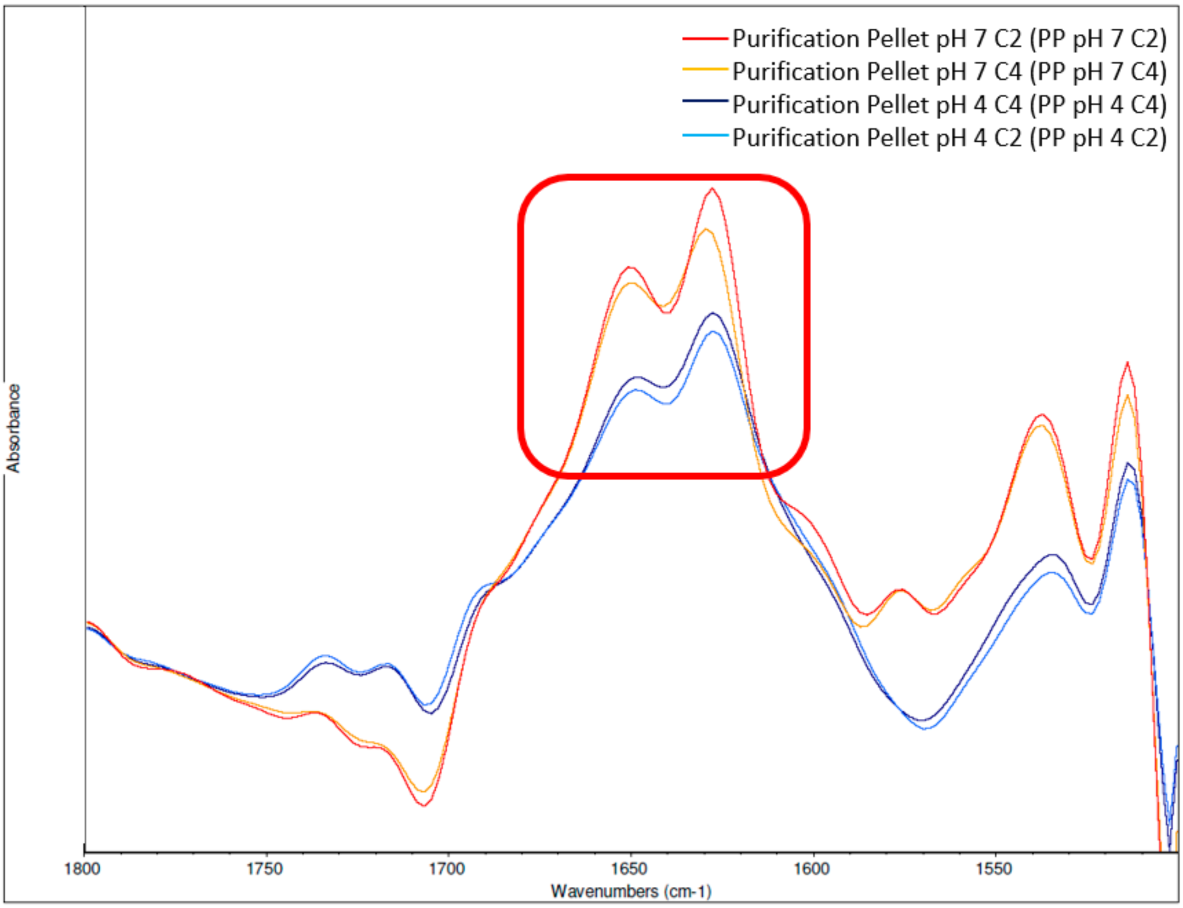

Figure S4: Fourier self-deconvoluted FTIR-ATR spectra zoomed in the 1800 cm^{-1} and 1500 cm^{-1} region of sample at pH 4 and pH 7 of the purification pellet (PP) and purification supernatant (PS) obtained from an initial powder concentration of 2% and 4% (C2 and C4 respectively).

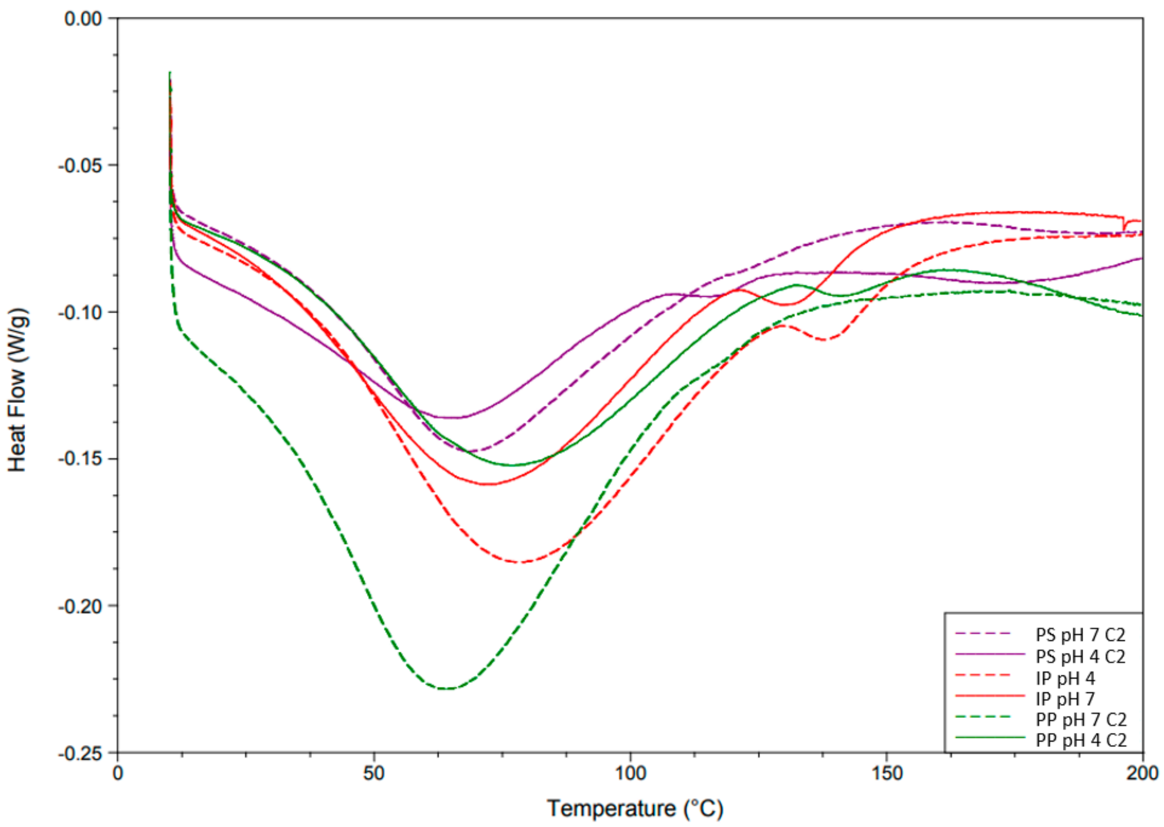

Figure S5: Representative Differential Scanning Calorimetry profiles of sample at pH 4 and pH 7 of the initial powder (IP), purification pellet (PP) and purification supernatant (PS) obtained from an initial powder concentration of 2% (C2). The curve areas were not normalized by protein concentration.
